# UTX inactivation in germinal center B cells promotes the development of multiple myeloma with extramedullary disease

**DOI:** 10.1038/s41375-023-01928-7

**Published:** 2023-05-17

**Authors:** Ola Rizq, Naoya Mimura, Motohiko Oshima, Shuji Momose, Naoya Takayama, Naoki Itokawa, Shuhei Koide, Asuka Shibamiya, Yurie Miyamoto-Nagai, Mohamed Rizk, Yaeko Nakajima-Takagi, Kazumasa Aoyama, Changshan Wang, Atsunori Saraya, Ryoji Ito, Masanori Seimiya, Mariko Watanabe, Satoshi Yamasaki, Tatsuhiro Shibata, Kiyoshi Yamaguchi, Yoichi Furukawa, Tetsuhiro Chiba, Emiko Sakaida, Chiaki Nakaseko, Jun-ichi Tamaru, Yu-Tzu Tai, Kenneth C. Anderson, Hiroaki Honda, Atsushi Iwama

**Affiliations:** 1 Division of Stem Cell and Molecular Medicine, Center for Stem Cell Biology and Regenerative Medicine, The Institute of Medical Science, The University of Tokyo, Tokyo, Japan; 2 Department of Cellular and Molecular Medicine, Graduate School of Medicine, Chiba University, Chiba, Japan; 3 Department of Hematology, Chiba University Hospital, Chiba, Japan; 4 Jerome Lipper Multiple Myeloma Center, Department of Medical Oncology, Dana-Farber Cancer Institute, Harvard Medical School, Boston, MA USA; 5 Department of Transfusion Medicine and Cell Therapy, Chiba University Hospital, Chiba, Japan; 6 Department of Pathology, Saitama Medical Center, Saitama Medical University, Kawagoe, Japan; 7 Department of Regenerative Medicine, Graduate School of Medicine, Chiba University, Chiba, Japan; 8 The State Key Laboratory of Reproductive Regulation and Breeding of Grassland Livestock, School of Life Sciences, Inner Mongolia University, Hohhot, China; 9 Central Institute for Experimental Animals, Kanagawa, Japan; 10 Department of Medical Technology and Sciences, School of Health Sciences at Narita, International University of Health and Welfare, Narita, Japan; 11 Department of Clinical Laboratory, Chiba University Hospital, Chiba, Japan; 12 Laboratory of Molecular Medicine, The Institute of Medical Science, The University of Tokyo, Tokyo, Japan; 13 Division of Clinical Genome Research, Advanced Clinical Research Center, The Institute of Medical Science, The University of Tokyo, Tokyo, Japan; 14 Department of Gastroenterology, Graduate School of Medicine, Chiba University, Chiba, Japan; 15 Department of Hematology, International University of Health and Welfare, Narita, Japan; 16 Field of Human Disease Models, Major in Advanced Life Sciences and Medicine, Institute of Laboratory Animals, Tokyo Women’s Medical University, Tokyo, Japan; 17 Laboratoty of Cellular and Molecular Chemistry, Graduate School of Pharmaceutical Sciences, The University of Tokyo, Tokyo, Japan

**Keywords:** Myeloma, Myeloma

## Abstract

UTX/KDM6A, a histone H3K27 demethylase and a key component of the COMPASS complex, is frequently lost or mutated in cancer; however, its tumor suppressor function remains largely uncharacterized in multiple myeloma (MM). Here, we show that the conditional deletion of the X-linked *Utx* in germinal center (GC) derived cells collaborates with the activating *Braf*^*V600E*^ mutation and promotes induction of lethal GC/post-GC B cell malignancies with MM-like plasma cell neoplasms being the most frequent. Mice that developed MM-like neoplasms showed expansion of clonal plasma cells in the bone marrow and extramedullary organs, serum M proteins, and anemia. Add-back of either wild-type *UTX* or a series of mutants revealed that cIDR domain, that forms phase-separated liquid condensates, is largely responsible for the catalytic activity-independent tumor suppressor function of UTX in MM cells. *Utx* loss in concert with *Braf*^*V600E*^ only slightly induced MM-like profiles of transcriptome, chromatin accessibility, and H3K27 acetylation, however, it allowed plasma cells to gradually undergo full transformation through activation of transcriptional networks specific to MM that induce high levels of *Myc* expression. Our results reveal a tumor suppressor function of UTX in MM and implicate its insufficiency in the transcriptional reprogramming of plasma cells in the pathogenesis of MM.

## Introduction

Deregulation of histone modifications has been attributed to an imbalance between writers that install the histone methylation marks and erasers that remove these marks [1]. Specifically, alteration of the methylation of lysine 27 on histone H3 (H3K27) has an incriminating role in hematological malignancies [2, 3]. The catalytic components of polycomb repressive complex 2 (PRC2), enhancer of zeste homolog 2 (EZH2) and its homolog EZH1, deposit the repressive H3K27me3 mark at both promoters and enhancers [4]. The histone demethylase, ubiquitously transcribed tetratricopeptide repeat gene on the X chromosome (UTX), also known as KDM6A, specifically removes di- and tri-methyl groups from H3K27 thus antagonizing PRC2-mediated transcriptional repression [5]. *UTX* was identified as a critical regulator of reprogramming in induced pluripotent stem cells (iPSCs) through its catalytic activity by partnering with Oct4, Sox2, and KIF4 reprogramming factors [6]. In addition to its H3K27 demethylase activity, UTX possesses catalytic-independent functions. It contributes to embryonic viability, differentiation, and certain aspects of development in mouse embryonic stem cells (ESCs) independently of its demethylase activity [7–9]. Importantly, UTX is an integral component of the MLL3 and MLL4 COMPASS (complex of proteins associated with Set1) complexes, which monomethylate H3K4 at enhancers [10]. UTX mediates the conversion of enhancers from an inactive to an active state by direct recruitment and coupling of p300/CBP histone acetyltransferases (HAT) and MLL4 [11].

Recent studies revealed that *UTX* is frequently mutated in different kinds of cancer, including hematological malignancies; however, the pathological role of *UTX* mutations in tumorigenesis is not yet fully understood [12]. *UTX* is often inactivated, exclusively in males, in T-cell acute lymphoblastic leukemia (T-ALL), where it functions as a tumor suppressor [13, 14]. UTX also suppresses the development of myeloid leukemia, in which it functions independently of its demethylase activity, through remodeling of enhancers and chromatin accessibility of oncogenes and tumor suppressor genes [15]. In multiple myeloma (MM), *UTX* inactivating mutations/deletions were detected in 1.5–4% of patients [16–18] and more frequently in established cell lines [19], suggesting a tumor suppressor role for UTX in MM and its implication in disease progression. Importantly, *UTX* inactivating lesions were associated with adverse overall survival [17]. *UTX*-null MM cell lines were more sensitive to EZH2 inhibitors compared to *UTX*-wild type (WT) MM [19].

To investigate the role of *UTX* insufficiency in the pathogenesis of MM, we generated a novel mouse model that had concurrent *Utx* loss and the activating *Braf*^*V600E*^ mutation. The activating mutations in the RAS/RAF/MEK/ERK/MAPK pathway are identified in up to 50% of newly diagnosed MM patients [16], and the serine-threonine kinase *BRAF* is mutated in 8-12% of MM patients at diagnosis, with V600E as the most common *BRAF* mutation [16, 20]. In this study, we demonstrated the tumor suppressor function of *Utx* in mature B cell malignancies including MM.

## Materials and methods

### Mice

All animal procedures were conducted in accordance with the Chiba University guidelines for the use of laboratory animals and approved by the Review Board for Animal Experiments of Chiba University (approval ID: 30-56) and Tokyo University (approval ID: PS18-02). Mice with conditional expression of *Braf*^*V600E*^ (LSL-*Braf* V600E) from its endogenous locus were previously described [21]. *Utx* conditional knockout mice were recently reported [22]. *Utx*^*fl*^*;Braf*^*V600E*^ mice were backcrossed at least 6 times onto a C57BL/6 (CD45.2) background and were crossed to *Cγ1-Cre* mice [23]. *Cγ1-Cre*-negative mice were used as controls. We immunized six- to eight-week-old mutant *Utx*/*Braf*, *Cγ1Cre+* and control mice using intraperitoneal injection of 50 µg NP-CGG (Biosearch Technologies) emulsified in complete Freund’s adjuvant (Sigma) and then 4–6 weeks later we gave them a second dose of NP-CGG in incomplete Freund’s adjuvant (Sigma). NOD.Cg-*Prkdc*^*scid*^*Il2rg*^*tm1Sug*^*/*Jic (NOG) and NOD.Cg-*Prkdc*^*scid*^*Il2rg*^*tm1Sug*^ Tg (CMV-IL6)/Jic (NOG-hIL-6) [24] mice were purchased from Central Institute for Experimental Animals (CIEA, Kawasaki, Japan). C57BL/6 mice congenic for the Ly5 locus (CD45.1) mice were purchased from Sankyo-Lab service, Tokyo, Japan.

## Results

### *Utx* loss cooperates with *Braf*^*V600E*^ to induce B-cell neoplasms

**Fig. 1:**
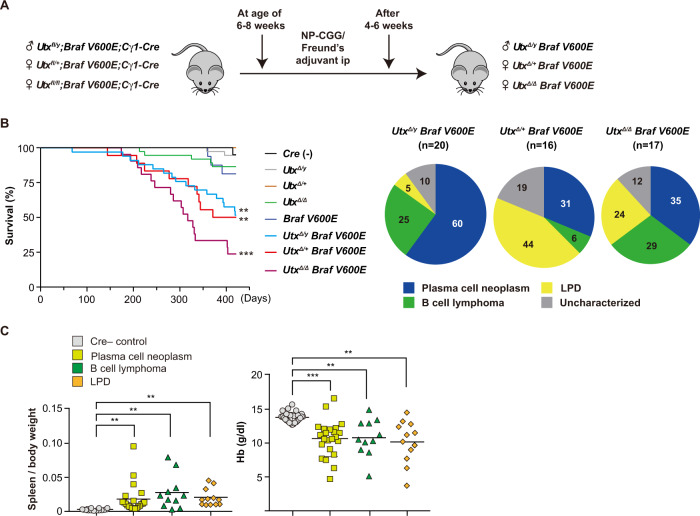
*Utx* insufficiency in association with mutant *Braf*^*V600E*^ induces mature B-cell neoplasms in mice. **A** Schematic diagram of experimental strategy. **B** Left panel: Kaplan–Meier survival analysis of *Cre* negative (*n* = 20), *Utx*^*Δ/y*^ (*n* = 37), *Utx*^*Δ/+*^ (*n* = 19), *Utx*^*Δ/Δ*^ (*n* = 37), *Braf*^*V600E*^ (*n* = 16), *Utx*^*Δ/y*^*Braf*^*V600E*^ (*n* = 33), *Utx*^*Δ/+*^*Braf*^*V600E*^ (*n* = 18), and *Utx*^*Δ/Δ*^*Braf*^*V600E*^ (*n* = 21) mice. Statistical significance of survival difference was determined by the log-rank test. **P < 0.01; and ***P < 0.001. Right panel: incidence of plasma cell neoplasms, B-cell lymphoma, and lymphoproliferative disease (LPD) in mice analyzed (*Utx*^*Δ/y*^*Braf*^*V600E*^, *n* = 20; *Utx*^*Δ/+*^*Braf*^*V600E*^, *n* = 16; *Utx*^*Δ/Δ*^ *Braf*^*V600E*^, *n* = 17). **C** Spleen weights normalized to body weight (left) and hemoglobin levels in PB (right) from mice that developed plasma cell neoplasms (PCNs, *n* = 21 and 23, respectively), B-cell lymphoma (BCL, *n* = 11 each) and lymphoproliferative disease (LPD, *n* = 12 each), and Cre-negative control mice (Cre-, *n* = 16 and 26, respectively). Bars indicate mean. **P < 0.01; ***P < 0.001 by student *t*-test.

To conditionally delete *Utx* in GC B cells, we crossed *Utx*^*fl*^ mice, in which exons 11 and 12 were floxed [22], with *Cγ1-Cre* mice, which express Cre recombinase in GC B cells, class switched memory B cells, and plasma cells in response to immunization [23]. The *Utx* allele that lacks exons 11 and 12 encoding a part of the TPR domain generates a frame shift mutant that fails to code for the C-terminal domains including the JmjC catalytic domain [22]. We crossed *Utx*^*fl*^ mice with *LSL-Braf*^*V600E*^ mice, in which the *Braf*^*V600E*^ allele is expressed from the endogenous *Braf* locus after Cre-mediated deletion of a lox-stop-lox (LSL) cassette [21]. We confirmed the efficient conditional deletion of *Utx* and LSL cassette using genomic polymerase chain reaction (PCR) of DNA extracted from spleen plasma cells (Supplementary Fig. 1A). *Utx* mRNA levels were significantly reduced in *Utx*-deficient plasma cells compared to control, possibly because of the activation of the nonsense-mediated mRNA decay pathway (Supplementary Fig. 1B). We hereafter refer to *Cγ1-Cre*;*Utx*^*fl/fl*^;*Braf*^*V600E/+*^, *Cγ1-Cre*;*Utx*^*fl/+*^;*Braf*^*V600E/+*^, and *Cγ1-Cre*;*Utx*^*fl/Y*^;*Braf*^*V600E/+*^ mice as *Utx*^*Δ/Δ*^*Braf*^*V600E*^, *Utx*^*Δ/+*^*Braf*^*V600E*^, and *Utx*^*Δ/Y*^*Braf*^*V600E*^, respectively (Fig. 1A).

During a long-term observation period, *Utx*^*Δ/+*^ females did not develop any lethal diseases, while *Utx*^*Δ/Δ*^ female and *Utx*^*Δ/Y*^ males developed lethal B cell malignancies, including plasma cell neoplasms, B-cell lymphoma, and lymphoproliferative disease (LPD) at low frequencies (Fig. 1B left panel and Supplementary Table 1). *Braf*^*V600E*^ mice also developed LPD and plasma cell neoplasms at low frequencies after a long latency (Fig. 1B left panel and Supplementary Table 1). Importantly, concurrent *Utx* loss and *Braf*^*V600E*^ expression significantly shortened the survival of mice compared to *Cre* negative control, single *Utx* loss, and single *Braf*^*V600E*^ expression (Fig. 1B left panel). *Utx*^*Δ/Δ*^*Braf*^*V600E*^ females succumbed to disease earlier than *Utx*^*Δ/Y*^*Braf*^*V600E*^ males and *Utx*^*Δ/+*^*Braf*^*V600E*^ females. Notably, *Utx*^*Δ/Δ*^*Braf*^*V600E*^*, Utx*^*Δ/Y*^*Braf*^*V600E*^, and *Utx*^*Δ/+*^*Braf*^*V600E*^ mice displayed heterogeneous phenotypes that included plasma cell neoplasms, B cell lymphoma, and LPD, with plasma cell neoplasms having the highest frequencies in *Utx*^*Δ/Δ*^*Braf*^*V600E*^ and *Utx*^*Δ/Y*^*Braf*^*V600E*^ mice (Fig. 1B right panel). Among the compound mice, *Utx*^*Δ/Y*^*Braf*^*V600E*^ male mice developed plasma cell neoplasms at a higher frequency than compound heterozygous and homozygous *Utx* females (Fig. 1B right panel). Sick mice showed splenomegaly and advanced anemia irrespective of the disease type (Fig. 1C).

### Development of B-cell lymphoma in *Utx* insufficient *Braf*^*V600E*^ mice

**Fig. 2:**
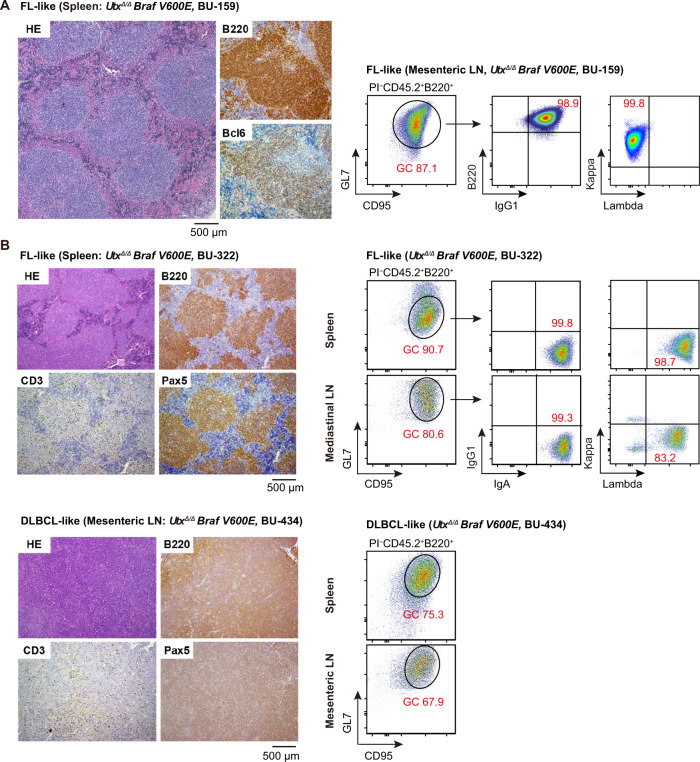
Development of B-cell lymphoma in *Utx* insufficient *Braf*^*V600E*^ mice. **A** Left panel: representative spleen sections harvested from a 40-week-old *Utx*^*Δ/Δ*^*Braf*^*V600E*^ mouse (BU-159) stained with hematoxylin and eosin or antibodies recognizing the indicated marker antigens. Right panel: representative plots of flow cytometric analysis of GC B cells in a mesenteric lymph node of the same mouse. Cells were first gated on live (PI^−^) CD45.2^+^ B220^+^ B-lymphocytes to determine the percentage of GC B cells (CD95^+^GL7^+^) and then we evaluated the clonality using IgG1, κ, and λ antibodies. **B** Left panel: representative spleen (upper) and mesenteric lymph node (LN) (lower) sections harvested from 45- and 40-week-old *Utx*^*Δ/Δ*^*Braf*^*V600E*^ mice (BU-322 and BU-434), respectively, stained with hematoxylin and eosin or antibodies recognizing the indicated marker antigens. Right panel: representative plots of flow cytometric analysis of GC B cells in the spleen and mediastinal or mesenteric lymph nodes of the same mice. We evaluated the clonality using IgA, κ, and λ antibodies.

A significant number of *Utx*^*Δ/Y*^*Braf*^*V600E*^, *Utx*^*Δ/+*^*Braf*^*V600E*^, and *Utx*^*Δ/Δ*^*Braf*^*V600E*^ mice developed B cell lymphoma with marked lymphadenopathy, splenomegaly, and hepatic involvement (25%, 6%, and 29%, respectively) (Fig. 1B right panel and Supplementary Table 2). Pathological examination of the spleen from *Utx*^*Δ/Δ*^*Braf*^*V600E*^ (BU-159) mouse revealed expansion of B220^+^BCL6^+^ GC B-lymphocytes, giving a picture resembling follicular lymphoma (FL) (Fig. 2A left panel). Flow cytometric analysis of a mesenteric LN from the same mouse confirmed clonal expansion of GC B cells (B220^+^CD95^+^GL7^+^) (Fig. 2A right panel). Immunohistochemical analyses of the spleen and lymph nodes (LNs) from BU-322 and BU-434 mice revealed loss of the normal architecture with follicular and diffuse expansion of neoplastic B220^+^Pax5^+^ B-lymphocytes, showing FL- and diffuse large B cell lymphoma (DLBCL)-like appearance, respectively (Fig. 2B left panel). Flow cytometric analysis confirmed the clonal B cell expansion in the spleen and enlarged LNs (Fig. 2B right panel). Detailed flow cytometric analysis of B-cell subsets in the spleens of lymphoma mice showed a decrease in the percentage of follicular B cells (FO; B220^+^CD23^+^CD21^low^) and an increase in transitional B cells (TR; B220^+^CD23^−^CD21^−^) or GC B cells (Supplementary Table 2).

While *UTX* mutations are rare in GC B-cell lymphomas [25], a high incidence of inactivating mutations in the components of COMPASS and its partner HATs, including *KMT2D*, *CREBBP*, and *EP300*, has been reported [26, 27]. Correspondingly, the deletion of *Kmt2d*, *Crebbp*, and *Ep300* in GC B cells has been found to promote lymphomagenesis in mice [28–30]. *UTX* deletion in Eμ-Myc mice not only accelerates lymphomagenesis, but also promotes tumor progression [31]. In addition, lymphomas with low *UTX* expression are associated with poor patient survival [31]. These findings suggest that *Utx* loss in GC B cells deregulates COMPASS/HAT, thereby promoting lymphomagenesis in mice. *BRAF* and *KRAS* mutations are identified relatively frequently (6.1 and 4.9%, respectively) in GCB type DLBCL [25]. Although a cooperative effect between COMPASS components and RAS-RAF cascade mutations has yet to be reported, our results suggest that their potential combined impact on the pathogenesis of GCB DLBCL is worth investigating.

### Development of plasma cell neoplasia in *Utx* insufficient *Braf*^*V600E*^ mice

**Fig. 3:**
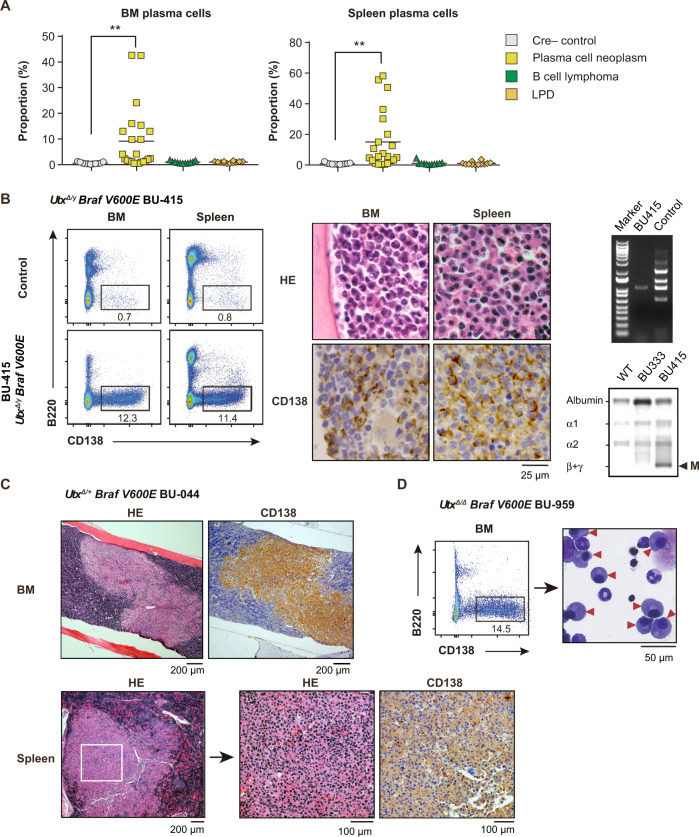
Development of plasma cell neoplasms in *Utx* insufficient *Braf*^*V600E*^ mice. **A** The percentage of plasma cells in the BM (left) and spleen (right) of mice that developed plasma cell neoplasms (BM, *n* = 23; spleen, *n* = 22), B-cell lymphoma (*n* = 11, each), LPD (*n* = 12, each), and Cre-negative control (*n* = 11, each). Bars indicate mean. **P < 0.01 by student *t*-test. **B** Left panel: representative plots of flow cytometric analysis of PI^−^ BM and spleen cells from a 38-week-old Cre-negative male control and 25-week-old *Utx*^*Δ/y*^*Braf*^*V600E*^ male mouse (BU-415). Proportions of plasma cells (CD138^+^B220^–^) are indicated. Middle panel: representative BM and spleen sections from BU-415 stained with hematoxylin and eosin or anti-CD138 antibody. Right panel: genomic PCR analysis of *Igh* rearrangement in BM plasma cells from a Cre-negative control and BU-415 (upper), and SPEP (Serum protein electrophoresis) performed on WT, BU333 (*Utx*^*Δ/+*^*Braf*^*V600E*^), and BU415 mice (lower). The positions of albumin and the different globulins are indicated. Arrowhead indicates an M-spike. **C** BM and spleen sections from BU-044 (*Utx*^*Δ/+*^*Braf*^*V600E*^) mouse stained with hematoxylin and eosin or anti-CD138 antibody. **D** Flow cytometric plot of PI^−^ BM cells from BU-959 (*Utx*^*Δ/Δ*^*Braf*^*V600E*^) (left) and cytological features of BM cells from BU-959 observed by Wright-Giemsa staining (right). Arrowheads indicate malignant plasma cells.

The most frequent neoplasm that developed in *Utx* insufficient *Braf*^*V600E*^ mice was plasma cell neoplasm: 60%, 31.3%, and 35.3% in *Utx*^*Δ/Y*^*Braf*^*V600E*^, *Utx*^*Δ/+*^*Braf*^*V600E*^, and *Utx*^*Δ/Δ*^*Braf*^*V600E*^ mice, respectively (Fig. 1B right panel). These mice showed significant increase in the percentage of plasma cells in the BM and spleen (Fig. 3A) and many of them had splenomegaly and advanced anemia (Fig. 1C). Flow cytometric analysis and immunohistochemistry revealed the diffuse infiltration of CD138^+^ plasma cells in the BM, spleen, LNs, and peritoneal cavity (Fig. 3B left and middle panels and Supplementary Table 3). Genomic PCR analysis of purified plasma cells revealed that they were of clonal origin showing a monotonous pattern of rearrangement of the immunoglobulin heavy chain (*Igh*) gene (Fig. 3B upper right panel). M-spike was detected in serum protein electrophoresis (SPEP) assays in half of the mice with plasma cell neoplasms (Fig. 3B lower right panel and Supplementary Table 3). Some mice showed focal expansion of plasma cells, mimicking plasmacytoma (Fig. 3C). BM and spleen plasma cells from moribund mice showed typical plasma cell morphology (Fig. 3B middle panel, C, and D). These results indicate that the mice with plasma cell neoplasms recapitulate the major characteristics of human multiple myeloma (MM), however, most of the plasma cell neoplasms involved extramedullary organs (Supplementary Table 3).

We next performed transplantation assays. We purified CD138^+^ plasma cells by magnetic column-based purification (average purity 70%). We then transplanted them into CD45.1 (Ly5.1) congenic B57BL/6 mice or NOD.Cg-*Prkdc*^*scid*^*Il2rg*^*tm1Sug*^*/*Jic (NOG) immunocompromised mice via tail vein (Supplementary Table 4). We also performed transplantation using bulk cells from BM and spleen. Most of the mice developed lethal LPD-like disease, but only a fraction of recipient mice showed evident expansion of malignant plasma cells (Supplementary Table 4), suggesting that the engraftment and expansion of clonal plasma cells were less efficient than polyclonal *Utx*-null post-GC B-cells.

### Modeling MM by using *Utx* insufficient myeloma cells

**Fig. 4:**
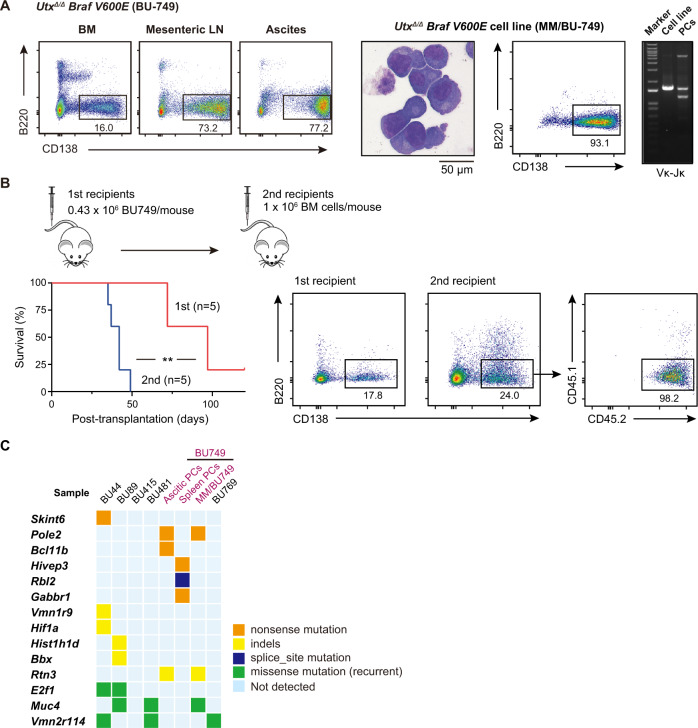
Modeling MM by using *Utx* insufficient myeloma cells. **A** Left panel: representative plots of flow cytometric analysis of BM, mesenteric LN, and ascites from a 42-week-old *Utx*^*Δ/Δ*^*Braf*^*V600E*^ female mouse (BU-749). Proportions of plasma cells (CD138^+^B220^−^PI^−^) are indicated. Right panel shows the characterization of an IL-6-dependent cell line derived from the plasmacytic ascites of BU-749. Cytological features observed by May-Giemsa staining (left), a representative plot of flow cytometric analysis of plasma cells (CD138^+^B220^−^PI^−^) (middle), and genomic PCR data of *Igl* Vκ-Jκ rearrangement (right) are depicted. Polyclonal spleen plasma cells (PCs) from control mice were used as a control of genomic PCR. **B** Upper panel: Schematic diagram of transplantation strategy. We injected 0.43 × 10^6^ BU749 cells, which were described in (**A**) and cultured in the presence of IL-6, into 5 sublethally irradiated (1.5 Gy) NOG mice. We recovered BM cells from moribund recipient mice and injected 1 × 10^6^ BM cells into secondary recipient NOG mice. Kaplan–Meier survival analysis of primary (*n* = 5) and secondary (*n* = 5) recipient mice is depicted (lower panel, left). Representative plots of flow cytometric analysis of BM from primary and secondary NOG recipient mice are shown (lower panel, right). Proportions of plasma cells (CD138^+^B220^−^PI^−^) are indicated. Statistical significance of survival difference was determined by the log-rank test. **P < 0.01. **C** A diagram summarizing 14 genes affected by somatic mutations detected in the indicated malignant plasma cells by whole exome sequencing. Non-recurrent missense mutations (139 genes) and the details of the samples are listed in Supplementary Table 5A–D.

To perform detailed characterization of *Utx* insufficient plasma cell neoplasms, we established several cell lines from plasmacytic ascites. One cell line was obtained from a moribund *Utx*^*Δ/Δ*^*Braf*^*V600E*^ mouse (BU-749, Supplementary Table 3) with an MM-like disease (Fig. 4A). The CD138^+^B220^−^ malignant plasma cells grew on TSt-4 stromal cells [32] in the presence of interleukin-6 (IL-6), and were of clonal origin showing monoclonal *Igl* gene rearrangement (Fig. 4A right panel). These cells, designated as MM/BU749, successfully engrafted in sublethally irradiated NOG mice, which subsequently developed deadly disease (Fig. 4B). Mice with advanced disease showed paralysis of the hindlimbs and body weight loss (data not shown). Importantly, the secondary recipients developed lethal disease much earlier than the primary recipients (Fig. 4B).

To understand the collaborating somatic mutations acquired by *Utx*-insufficient *Braf*^*V600E*^ plasma cell clones during disease initiation and progression, we performed whole exome sequencing (WES) using DNA of plasma cells purified from BM, spleen, and ascitic fluid of mice with plasma cell neoplasms and MM/BU749 cell line. DNA of corresponding tail or PB B cells was used as a control (Supplementary Table 5A). The mean depth of WES was 50X and the mean coverage of target sequences with an average depth of more than 10X was greater than 95% (Supplementary Tables 5A and B). A total of 153 somatic mutations were identified in 112 genes in 8 samples (Fig. 4C and Supplementary Tables 5C and D). Missense mutations were recurrently detected in at least 2 cases, in only three genes: *E2f1*, *Muc4*, and *Vmn2r114*. Other mutations included 5 nonsense mutations, 5 frameshift indels, 1 splice-site mutation, and 139 missense mutations (Fig. 4C and Supplementary Tables 5C and D). Unexpectedly, none of these mutations were major driver mutations in MM [16].

UTY is the Y chromosome homolog of UTX with weaker tumor-suppressive activity compared to UTX [33]. It has been reported that *Uty* expression protects *Utx*^*-/Y*^ male mice from leukemogenesis [15]. In addition, *UTY* is frequently lost or mutated in pancreatic tumors with squamous differentiation in male patients [34]. Genomic loss of *UTY* in male cancer cell lines with inactivating *UTX* mutations (13/16, 81%) is significantly more frequent than *UTY* loss in *UTX* wild-type cancers (153/307, 49%) [18]. These findings support a tumor suppressive function of *UTY* in a setting of *UTX* insufficiency. However, our WES for male mice that developed MM-like disease did not show any abnormalities in *Uty* or Y-chromosome. Of interest, the mutation profiles of splenic and ascitic plasma cells from BU749 mouse were totally different, indicating the clonal heterogeneity of malignant plasma cells in the same mouse. These results indicate that *Utx* loss and *Braf*^*V600E*^ are the main drivers of tumorigenesis in this MM model.

### Catalytic activity is dispensable for the tumor suppressor function of UTX in MM

**Fig. 5:**
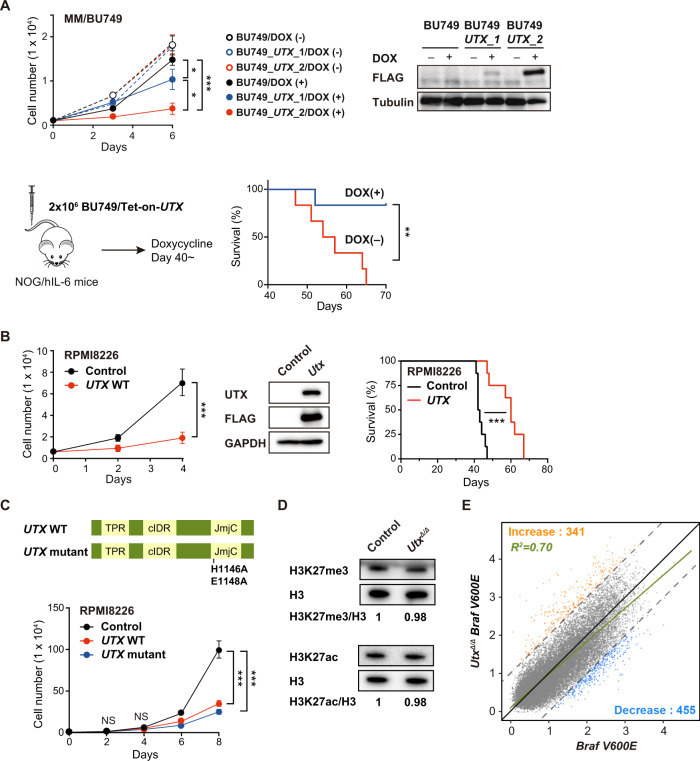
Catalytic activity is dispensable for the tumor suppressor function of UTX. **A** Effects of *UTX* add-back on *Utx*-null BU749 cell growth. Upper left panel: Growth of BU749 with DOX-regulated human *UTX* in the presence and absence of DOX (2 μg/ml). Upper right panel: Expression of Flag-UTX protein was detected by western blotting with anti-Flag antibody. α-tubulin served as a loading control. Lower left panel: schematic diagram of transplantation strategy. Lower right panel: Kaplan–Meier survival analysis of sublethally (1.5 Gy) irradiated NOG-hIL-6 mice (*n* = 6, each) receiving 2 million BU749 cells per mouse. DOX was given to the mice in drinking water from day 40 post-transplantation and the mice were observed for 30 days. DOX dose: 0.2% (2 mg/mL) in drinking water, containing 3.5% sucrose, to be changed twice a week and protected from light. **B** Effects of *UTX* add-back on *UTX*-null human MM RPMI8226 cell growth. Left panel: growth of *UTX*-null human RPMI8226 cells transduced with the indicated lentiviruses. Middle panel: expression of Flag-UTX protein was detected by western blotting with anti-UTX and Flag antibodies. GAPDH served as a loading control. Right panel: Kaplan–Meier survival analysis of sublethally (1.5 Gy) irradiated NOG-hIL-6 mice receiving 3 million RPMI8226 cells transduced with either control (*n* = 8) or *UTX* (*n* = 8) lentivirus per mouse. **C** Upper panel: schematic representation of WT and demethylase inactive mutant UTX. Lower panel: effects of WT and demethylase inactive mutant *UTX* add-back on *UTX*-null human MM RPMI8226 cell growth. **D** Western blot analyses of global histone modification levels in plasma cells from control and *Utx*^*Δ/Δ*^ mice using anti-H3K27me3, H3K27ac, and histone H3 antibodies. Levels of H3K27me3 and H3K27ac were normalized to the amount of H3 and are indicated relative to wild-type control values at the bottom. **E** Scatterplot showing the relationship of the fold enrichment values of H3K27me3 ChIP signals against the input signals (ChIP/input) at TSS ± 2.0 kb of RefSeq genes between *Braf*^V600E^ and *Utx*^*Δ/Δ*^*Braf*^*V600E*^ mice. The dotted light gray lines represent the boundaries for twofold increase and twofold decrease, respectively. The genes that gained and lost H3K27me3 levels greater than twofold in *Utx*^*Δ/Δ*^*Braf*^*V600E*^ plasma cells compared with *Braf*^*V600E*^ plasma cells are indicated in orange and blue, respectively. Cell growth data are shown as the mean ± SD of triplicate cultures. Statistical significance of survival difference and cell growth was determined by the log-rank test and the student *t*-test, respectively. *P < 0.05; **P < 0.01; ***P < 0.001.

Making use of our *Utx*-deficient plasma cell line, we evaluated the impact of *UTX* add-back on *Utx*-null MM/BU749 cell growth. We conditionally overexpressed human *UTX* in MM/BU749 cells using a Tet-on lentivirus system. Exogenous *UTX* significantly impaired the proliferation of MM/BU749 in suspension culture in the presence of doxycycline (DOX) (Fig. 5A upper left panel). Growth suppressive effects of *UTX* add-back correlated well with the expression levels of exogenous *UTX* (Fig. 5A upper right panel). We next transplanted MM/BU749 cells with DOX-regulated exogenous *UTX* into NOG mice. In this experiment, we used NOD.Cg-*Prkdc*^*scid*^*Il2rg*^*tm1Sug*^ Tg (CMV-IL6)/Jic (NOG-hIL-6) mice [24] to facilitate plasma cell engraftment. Induction of *UTX* in MM/BU749 clearly prevented the development of lethal disease in recipient mice and prolonged their survival (Fig. 5A lower panels), confirming the deleterious effects of *UTX* add-back on *Utx*-null plasma cell expansion in vivo. *UTX* add-back in *UTX*-null human MM cell line (RPMI8226) also impaired their growth (Fig. 5B left panel) and prolonged the survival of recipient mice in xenotransplantation assays (Fig. 5B right panel) as previously reported [19].

To investigate the role of the demethylase activity of UTX in MM, we transduced RPMI8226 cells with WT and a demethylase-inactive mutant *UTX*. Point mutations at amino acid residues of the Fe(II)-binding motif in the JmjC domain (H1146 and E1148) abolish the demethylase activity of UTX (Fig. 5C upper panel) [35, 36]. Of note, add-back of H1146A/E1148A mutant impaired the growth of RPMI8226 cells in a manner similar to WT UTX (Fig. 5C lower panel). We then performed western blot analysis of histone modifications in plasma cells from *Utx*^*Δ/Δ*^ mice and found no significant changes in both H3K27me3 and H3K27ac levels (Fig. 5D). In addition, ChIP-seq analysis did not reveal any significant change in H3K27me3 levels in *Utx*^*Δ/Δ*^*Braf*^*V600E*^ plasma cells compared to *Braf*^*V600E*^ plasma cells (Fig. 5E). These results indicate that UTX exerts a tumor suppressor function in a demethylase activity-independent manner.

Recently, it has been proposed that the tumor suppressor function of UTX largely relies on UTX condensation, higher-order assembles that are mediated by a core intrinsically disordered region (cIDR) [33]. We therefore tested the add-back of ΔcIDR UTX mutant in *UTX*-null MM cells. A UTX mutant lacking cIDR mostly failed to suppress the growth of the cells (Supplementary Fig. 2A, B). These results suggest that the cIDR domain is largely responsible for the tumor suppressor function of UTX in MM.

### *Utx* loss with *Braf*^*V600E*^ induces myeloma-like gene signature in plasma cells

**Fig. 6:**
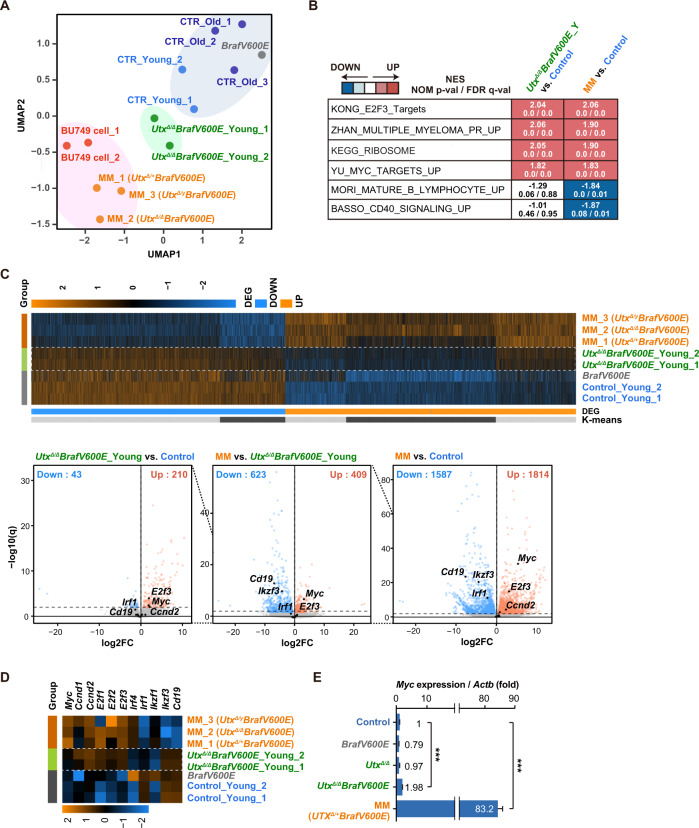
*Utx* loss induces myeloma-like gene signature in plasma cells. **A** UMAP plot based on the z-scores of expression values (DESeq2 normalized counts) of RNA-seq data of plasma cells from young (21 ~ 22-week-old) and old (40 ~ 55-week-old) control, *Braf*^*V600E*^ (31-week-old), young *Utx*^*Δ/Δ*^*Braf*^*V600E*^ (20-week-old) mice, compound mice with overt MM (MM1-3, 25 ~ 52-week-old), and BU749 (*Utx*^*Δ/Δ*^*Braf*^*V600E*^) cells. **B** GSEA using RNA-seq data of BM plasma cells from the indicated mice. Summary of GSEA data of representative gene sets is shown. Normalized enrichment scores (NES), nominal *p* values (NOM), and false discovery rates (FDR) are indicated. The GSEA data are listed in Supplementary Table 6A and B. **C** Upper panel; heatmap showing the *z*-scores of expression values (DESeq2 normalized counts) of DEGs (FDR *q* < 0.01) in BM plasma cells from compound mice with overt MM, *Braf*^*V600E*^ and young *Utx*^*Δ/Δ*^*Braf*^*V600E*^ mice in comparison with control plasma cells. Lower panel: volcano plots of DEGs normalized by DESeq2. Genes up-regulated and down-regulated are shown in red and blue, respectively. **D** Heatmap showing the *z*-scores of expression values (DESeq2 normalized counts) of representative myeloma signature genes. **E** Quantitative RT-PCR analysis of *Myc* in BM plasma cells from control, *Braf*^*V600E*^, *Utx*^*Δ/Δ*^, young *Utx*^*Δ/Δ*^*Braf*^*V600E*^, and MM *Utx*^*Δ/+*^*Braf*^*V600E*^ mice. *Actb* was used to normalize the amount of input RNA. Data are shown as the mean ± SEM of triplicates. ***P < 0.001 by student *t*-test.

To understand the impact of *UTX* loss on the plasma cell transcriptome, we performed RNA-seq of plasma cells from young (20 ~ 25-week-old) and old (40 ~ 50-week-old) *Cγ1-Cre* negative control mice, *Braf*^*V600E*^ mice (31-week-old), young *Utx*^*Δ/Δ*^*Braf*^*V600E*^ mice (20 ~ 25-week-old), *Utx/Braf*^*V600E*^ mice with overt MM (MM1-3, 20 ~ 50-week-old), and BU749 (*Utx*^*Δ/Δ*^*Braf*^*V600E*^) cells. A Uniform Manifold Approximation and Projection (UMAP) analysis revealed that *Utx* loss together with *Braf*^*V600E*^ readily induced moderate transcriptomic reprogramming of plasma cells towards MM-like transcriptome (Fig. 6A). Gene set enrichment analysis revealed activation of gene sets associated with cell cycle, Myc targets, and ribosome biogenesis and downregulation of gene sets associated with B lymphocytes and CD40 signal in *Utx/Braf*^*V600E*^ mice (Fig. 6B). The change in the expression levels of these gene sets was moderate in *Utx*^*Δ/Δ*^*Braf*^*V600E*^ mice without overt MM compared with those in *Utx/Braf*^*V600E*^ mice with overt MM (Supplementary Fig. 3, Supplementary Tables 6A and B). We then defined differentially expressed genes (DEGs) between young *Utx*^*Δ/Δ*^*Braf*^*V600E*^, MM, and control plasma cells (FDR *q* < 0.01, Supplementary Table 7). *Utx/Braf*^*V600E*^ plasma cells before and after overt MM showed gradual up-regulation and down-regulation of DEGs (Fig. 6C upper panel), including representative myeloma signature genes with upregulation of *Myc*, *Ccnd2*, *E2f3*, and *Irf4* and downregulation of *Cd19* and *Ikzf3* (Fig. 6C lower panel, D). *MYC* is one of the key genes in the pathogenesis of multiple myeloma. Of interest, neither *Braf*^*V600E*^ nor *Utx*^*Δ/Δ*^ mice showed *Myc* activation in plasma cells. Intriguingly, *Utx*^*Δ/Δ*^*Braf*^*V600E*^ mice before overt MM showed very mild albeit significant up-regulation of *Myc* compared to a mouse with overt MM (Fig. 6E). These results indicate that the myeloma-like transcriptional landscape develops gradually in *Utx/Braf*^*V600E*^ plasma cells leading to growth advantage in vivo.

### Characteristics of chromatin accessibility in *Utx*-null myeloma cells

**Fig. 7:**
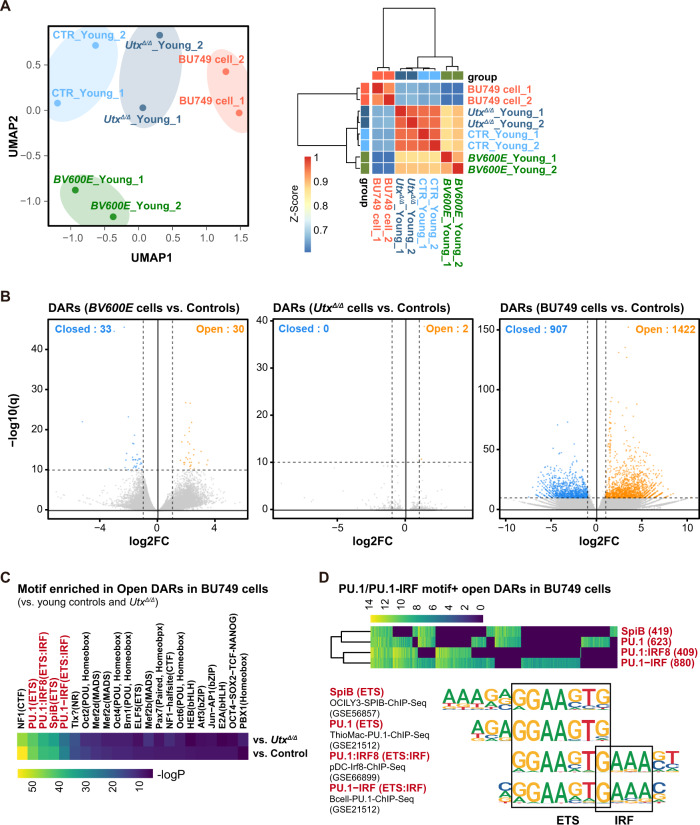
Characteristics of chromatin accessibility in *Utx*-null myeloma cells. **A** Left panel: UMAP plot based on ATAC peak profiling of plasma cells from young control, *Braf*^*V600E*^, *Utx*^*Δ/Δ*^ mice (24–27-week-old), and BU749 (*Utx*^*Δ/Δ*^*Braf*^*V600E*^) plasma cells. Right panel: hierarchical clustering based on the Pearson’s correlation coefficient of ATAC peaks. **B** Volcano plots of DARs in *Braf*^*V600E*^ and *Utx*^*Δ/Δ*^ plasma cells and BU749 cells compared with young control plasma cells (*p* < 10^−10^, log2FC > 1). **C** Heat map showing the enrichment of the transcription factor binding motifs at open DARs in BU749 cells using hypergeometric distribution of Homer software. The combined peaks detected in all samples were used for the background to remove imbalance in the sequence content. **D** Heat map showing the enrichment of the indicated transcription factor binding motifs at each open DAR in BU749 cells calculated as in (**C**).

To understand the transcriptional networks operating in *Utx*-null myeloma cells, we performed ATAC-seq in plasma cells from young *Cγ1-Cre* negative control, *Braf*^*V600E*^ and *Utx*^*Δ/Δ*^ mice (24-27-week-old), and BU749 (*Utx*^*Δ/Δ*^*Braf*^*V600E*^) plasma cells. UMAP analysis and hierarchical clustering of ATAC-seq data revealed that BU749 cells had largely different chromatin accessibility from control, *Braf*^*V600E*^, and *Utx*^*Δ/Δ*^ plasma cells, while the changes among control, *Braf*^*V600E*^, and *Utx*^*Δ/Δ*^ plasma cells were mild (Fig. 7A). We next defined differentially accessible regions (DARs) between control plasma cells and BU749 cells using DEseq2 and biological duplicates (*n* = 2). We found that 1422 and 907 DARs were open and closed in BU749 cells (*p* < 10^−10^, log2FC (fold change) >1), respectively (Fig. 7B, Supplementary Table 8). The majority of DARs were localized at the intergenic or intron regions that generally represent active or poised enhancers, or promoter regions. (Supplementary Fig. 4A). However, only a few DARs were detected that matched the strict criteria of *p* < 10^−10^, log2FC > 1 in *Braf*^*V600E*^ and *Utx*^*Δ/Δ*^ plasma cells (Fig. 7B).

We then compared the frequencies of the transcription factor binding motifs at open DARs in BU749 cells using hypergeometric distribution of Homer software and calculated the *p*-values of enrichment. The combined peaks detected in all samples were used for the background to remove imbalance in the sequence content. This motif analysis revealed that the binding motifs of PU.1-IRF, PU.1-IRF8, PU.1, and SpiB were highly enriched in open DARs in BU749 cells (Fig. 7C and Supplementary Fig. 4B). PU.1-IRF motif was most frequently identified in open DARs in BU749 compared with control plasma cells (Fig. 7D). Importantly, *Irf4* was one of the *Irf* genes with high expression in MM mice cells. In addition, *Spi1* encoding Pu.1 was also highly expressed in MM cells (Supplementary Fig. 4C). The transcription factor IRF4 (interferon regulatory factor 4) is required for the development, maintenance, and function of plasma cells [37–39]. IRF4 is also a key regulator of multiple myeloma and directly activates *MYC* in myeloma cells [40]. IRF4 binds with low affinity to interferon sequence response elements (ISREs). In contrast, it binds with high affinity to ETS-IRF composite motifs (EICE) through interaction with the transcription factors PU.1 or SpiB [41, 42]. In addition, IRF4 also cooperatively assembles with BATF on composite AP-1-IRF (AICE) motifs [43]. To assess the correlation between changes in chromatin accessibility and transcription, DARs were connected to the nearest genes based on their distance to TSSs. The genes linked to open DARs, including those linked to DARs with PU.1-IRF motif, showed significantly higher levels of expression in BU749 cells than control plasma cells (Supplementary Fig. 4D).

**Fig. 8:**
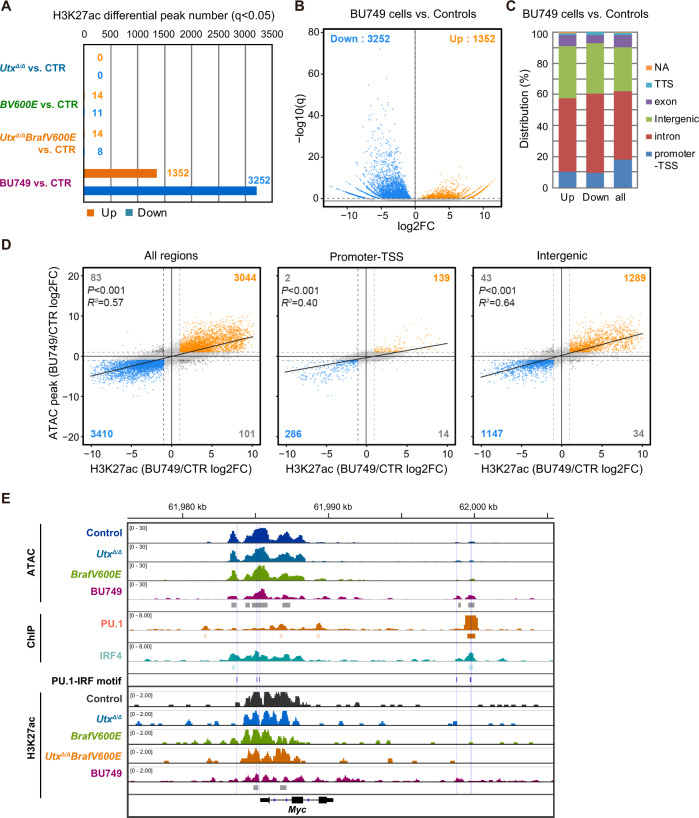
Relationship between chromatin accessibility, H3K27 acetylation and transcription in *Utx*-null myeloma cells. **A** Number of differential H3K27ac peaks in *Braf*^*V600E*^, *Utx*^*Δ/Δ*^, and *Utx*^*Δ/Δ*^*Braf*^*V600E*^ plasma cells and BU749 cells compared to control. **B** Volcano plot of differential H3K27ac peaks in BU749 cells compared with control plasma cells (*q* < 0.05). **C** Percentage of each genomic annotation of H3K27ac peaks. **D** Correlation of H3K27ac and ATAC peaks in all genomic regions, promoter-TSS, and intergenic regions. *P*, *p*-value and R2, adjusted R-squared of the regression model. **E** Snapshots of ATAC-seq signals and H3K27ac modification at the *Myc* locus in control, *Braf*^*V600E*^, *Utx*^*Δ/Δ*^ and *Utx*^*Δ/Δ*^*Braf*^*V600E*^ plasma cells, and BU749 cells. The localization of PU.1-IRF motifs is indicated. The ChIP peaks of PU.1 and IRF4 in plasmablasts [44] are also depicted.

To understand the epigenomic status of DARs, we profiled the acetylation status of H3K27 (H3K27ac) in plasma cells from control, *Braf*^*V600E*^, *Utx*^*Δ/Δ*^, and *Utx*^*Δ/Δ*^*Braf*^*V600E*^ mice and BU749 (*Utx*^*Δ/Δ*^*Braf*^*V600E*^) plasma cells. The H3K27ac status followed a similar pattern to the chromatin accessibility profile, only a few differential H3K27ac peaks were detected in *Braf*^*V600E*^, *Utx*^*Δ/Δ*^, and *Utx*^*Δ/Δ*^*Braf*^*V600E*^ plasma cells compared to control (Fig. 8A). In contrast, we found that the read counts of 1,352 and 3,252 peaks were increased and decreased in BU749 cells (*q* < 0.05), respectively (Fig. 8B, Supplementary Table 9). The majority of differential H3K27ac peaks were localized at the intergenic or intron regions that generally represent active or poised enhancers, or promoter regions. (Fig. 8C). The status of H3K27ac modification was well correlated with that of chromatin accessibility, particularly in the intergenic regions (Fig. 8D).

We then compared open DARs in BU749 cells with the published ChIP-seq data of IRF4 and PU.1 in plasmablasts [44]. As expected, many of the open DARs with PU.1-IRF motif overlapped with the IRF4 and PU.1 ChIP peaks in plasmablasts (Supplementary Fig. 4E). PU.1-IRF motif was identified at the *Myc* locus (Fig. 8E), a direct target of IRF4 [40]. However, chromatin accessibility at the *Myc* locus appeared to be already open with the presence of H3K27 acetylation in control plasma cells and showed no significant changes in *Utx*^*Δ/Δ*^ plasma cells (Fig. 8E). This was in line with the insignificant change in *Myc* expression observed in *Utx*^*Δ/Δ*^ plasma cells (Fig. 6E). In contrast, BU749 cells showed increased chromatin accessibility with the presence of H3K27 acetylation, particularly at +13 kb of the *Myc* locus that contained PU.1 and IRF4 binding sites (Fig. 8E). These data suggest that epigenomic reprogramming gradually proceeds in the absence of UTX and plasma cell clones that acquire myeloma-like properties are selected over time.

### Sensitivity of *UTX* insufficient cells to pharmacological inhibition

*UTX* inactivating lesions in MM were associated with adverse overall survival, suggesting the association of *UTX* insufficiency with resistance to conventional therapy [17]. We therefore investigated the susceptibility of UTX insufficient myeloma cell lines to a proteasome inhibitor, bortezomib (BTZ); an immunomodulatory drug, lenalidomide; and a bromodomain inhibitor, JQ1. *UTX* insufficient cell lines (RPMI8226 and U266) required nearly a twofold higher concentration of BTZ to inhibit cell viability by 50% (cytotoxic concentration; CC_50_). Of note, *UTX* insufficient cell lines were highly resistant to lenalidomide and JQ1 compared with MM cell lines with WT UTX (Supplementary Fig. 5).

## Discussion

In this study, we investigated the impact of conditional deletion of *Utx* in germinal center and post-germinal center B cells and plasma cells. Conditional deletion of *Utx* in a *Braf*^*V600E*^ setting induced mature B cell malignancies including B cell lymphomas and multiple myeloma with the shortest survival being observed in *Utx*^*Δ/Δ*^*Braf*^*V600E*^ mice. Our mouse model recapitulated the cardinal features of plasma cell neoplasms such as increased percentage of plasma cells in the BM, anemia, and large M spikes in SPEP. The main limitation was the absence of myeloma-like lytic bone lesions. Importantly, we did not find any major MM driver gene mutations or mutations in *Uty* in MM cells from moribund mice by WES. This well corresponded to the murine *Utx*^*Δ/Δ*^ acute myeloid leukemia data that showed no recurrently mutated genes, with the exception of *Skint11* (two of seven samples) [15]. These findings indicate that *UTX* insufficiency is a strong driver of myeloma development. Re-expression of *UTX* in both our *Utx*-deficient murine cell line (BU749) and human UTX-null RPMI8226 cells significantly inhibited the growth of the cells in vitro and in vivo. This is in agreement with the observations in human MM cell lines by *Ezponda et al*. [19]. Importantly, an enzymatically inactive UTX variant also suppressed the growth of UTX-null human RPMI8226 cells and this together with the insignificant change in H3K27me3 in *Utx*^*Δ/Δ*^*Braf*^*V600E*^ plasma cells compared to control by ChIP-seq, suggests that the demethylase activity is dispensable for the tumor suppressor function of UTX in MM. While the H3K27-demethylase activity of UTX is thought to be essential for its tumor suppressor role in T-ALL [13, 14], it is deemed to be redundant in acute myeloid leukemia and pancreatic cancer [15, 34]. Recently, Shi *et al*. [33] reported that the tumor suppressor function of UTX largely relies on the TPR domain and the ability to form condensates via cIDR. Our results confirmed the essential role of the cIDR domain in the tumor suppressor activity of UTX in MM cells. In addition to the *UTX* inactivating mutations/deletions found in MM, inactivating mutations have also been detected in the components of COMPASS and its partner HATs, including *KMT2C*, *CREBBP*, and *EP300* [16], further implicating dysregulated COMPASS activity in MM. Interestingly, the cooperativity between *Utx* loss and *Braf*^V600E^ mutation in our mouse model points out the probable synergism between mutations in the COMPASS components and activation of the RAS-RAF-MEK-ERK/MAPK cascade in MM.

UTY has weaker tumor-suppressive activity than UTX. However, it functions as a tumor suppressor in a setting of *UTX* insufficiency [15, 18, 34]. Our WES for male MM mice did not show any abnormalities in *Uty* or Y-chromosome. The residual Uty function in *Utx*^*Δ/Y*^*Braf*^*V600E*^ males may explain the histological bias observed between male and female compound mice and the fact that *Utx*^*Δ/Δ*^*Braf*^*V600E*^ females developed disease earlier than *Utx*^*Δ/Y*^*Braf*^*V600E*^ males. It would be intriguing to explore the role of Uty in this mouse model.

Our RNA-seq analysis revealed that the oncogene *Myc* was dramatically upregulated in *Utx*^*Δ/Δ*^*Braf*^*V600E*^ MM cells but not in *Utx*^*Δ/Δ*^ and *Utx*^*Δ/Δ*^*Braf*^*V600E*^ plasma cells at early time points post-*Utx* deletion. Activation of *MYC* is common in MM [45] and overexpression of *Myc* in late B cells induced MM-like disease in mice [46]. In addition, *Irf4*, which is essential for the survival of MM cells [40], was also upregulated in mice with MM-like disease. Importantly, an autoregulatory loop between *IRF4* and *MYC* has been described in MM [40]. Although GSEA revealed that gene sets related to *Myc*, multiple myeloma, and cell cycle were activated in young *Utx*^*Δ/Δ*^*Braf*^*V600E*^ mice, the changes appeared to be very mild. Consistently, ATAC-seq and CUT&TAG data indicated very mild changes in chromatin accessibility and H3K27ac modification in *Utx*^*Δ/Δ*^ plasma cells at an early time point post-*Utx* deletion. The PU.1-IRF motif most highly enriched in open DARs in *Utx*^*Δ/Δ*^*Braf*^*V600E*^ MM cells was not enriched in open DARs in *Utx*^*Δ/Δ*^ plasma cells. These results suggest that while UTX loss does not immediately induce drastic phenotypic changes in plasma cells, it allows them to undergo gradual genome-wide re-organization and transcriptional reprogramming. Subsequently, plasma cell clones that acquired MM-like properties are selected over time. This process could involve re-organization of epigenetic regulators such as histone modifiers, as we observed changes in chromatin accessibility and H3K27ac at the *Myc* locus. Alternatively, it is possible that UTX loss induces severe phenotypic changes in selected plasma cells, which expand to overt MM over time. Further analyses are needed to clarify the mechanism of myelomagenesis in our mouse model.

MM patients with a UTX deletion or mutation have a worse overall survival (OS) compared with those with WT UTX [17]. We confirmed that *UTX* insufficient cell lines were highly resistant to lenalidomide and JQ1 compared with MM cell lines with WT UTX. Bromodomain inhibitors negatively regulates promoter and enhancer activity, including those of well-known oncogenic transcription factor genes such as *MYC* and *FOSL1* [47–49]. This suggests that *UTX* insufficiency induces enhancer reorganization at critical oncogenic gene loci and stabilizes their active enhanceosome. This is consistent with the marked activation of *MYC* in *UTX* insufficient myeloma cells in our MM mouse model.

In summary, we established a novel myeloma mouse model in which *Utx* loss and *Braf*^*V600E*^ are combined. This study clearly demonstrates a catalytic activity-independent tumor suppressor function of UTX in MM and implicates its insufficiency in the transcriptional reprogramming of plasma cells. Our mouse model could be a useful tool for understanding the role of epigenetic dysfunction in mature B cell malignancies and studying novel therapeutic agents for MM.

## Supplementary information

Supplementary Information

Supplementary Table 1-9

## Data Availability

RNA- and ChIP-sequencing data and CUT&TAG data obtained in this study were deposited in DNA Data Bank of Japan (DDBJ) (accession numbers DRA13765 and DRA015180). A complete and detailed description of methods is provided in Supplementary Methods.
