# Correction: UTX inactivation in germinal center B cells promotes the development of multiple myeloma with extramedullary disease

**DOI:** 10.1038/s41375-023-01969-y

**Published:** 2023-07-18

**Authors:** Ola Rizq, Naoya Mimura, Motohiko Oshima, Shuji Momose, Naoya Takayama, Naoki Itokawa, Shuhei Koide, Asuka Shibamiya, Yurie Miyamoto-Nagai, Mohamed Rizk, Yaeko Nakajima-Takagi, Kazumasa Aoyama, Changshan Wang, Atsunori Saraya, Ryoji Ito, Masanori Seimiya, Mariko Watanabe, Satoshi Yamasaki, Tatsuhiro Shibata, Kiyoshi Yamaguchi, Yoichi Furukawa, Tetsuhiro Chiba, Emiko Sakaida, Chiaki Nakaseko, Jun-ichi Tamaru, Yu-Tzu Tai, Kenneth C. Anderson, Hiroaki Honda, Atsushi Iwama

**Affiliations:** 1 Division of Stem Cell and Molecular Medicine, Center for Stem Cell Biology and Regenerative Medicine, The Institute of Medical Science, The University of Tokyo, Tokyo, Japan; 2 Department of Cellular and Molecular Medicine, Graduate School of Medicine, Chiba University, Chiba, Japan; 3 Department of Hematology, Chiba University Hospital, Chiba, Japan; 4 Jerome Lipper Multiple Myeloma Center, Department of Medical Oncology, Dana-Farber Cancer Institute, Harvard Medical School, Boston, MA USA; 5 Department of Transfusion Medicine and Cell Therapy, Chiba University Hospital, Chiba, Japan; 6 Department of Pathology, Saitama Medical Center, Saitama Medical University, Kawagoe, Japan; 7 Department of Regenerative Medicine, Graduate School of Medicine, Chiba University, Chiba, Japan; 8 The State Key Laboratory of Reproductive Regulation and Breeding of Grassland Livestock, School of Life Sciences, Inner Mongolia University, Hohhot, China; 9 Central Institute for Experimental Animals, Kanagawa, Japan; 10 Department of Medical Technology and Sciences, School of Health Sciences at Narita, International University of Health and Welfare, Narita, Japan; 11 Department of Clinical Laboratory, Chiba University Hospital, Chiba, Japan; 12 Laboratory of Molecular Medicine, The Institute of Medical Science, The University of Tokyo, Tokyo, Japan; 13 Division of Clinical Genome Research, Advanced Clinical Research Center, The Institute of Medical Science, The University of Tokyo, Tokyo, Japan; 14 Department of Gastroenterology, Graduate School of Medicine, Chiba University, Chiba, Japan; 15 Department of Hematology, International University of Health and Welfare, Narita, Japan; 16 Field of Human Disease Models, Major in Advanced Life Sciences and Medicine, Institute of Laboratory Animals, Tokyo Women’s Medical University, Tokyo, Japan; 17 Laboratoty of Cellular and Molecular Chemistry, Graduate School of Pharmaceutical Sciences, The University of Tokyo, Tokyo, Japan

**Keywords:** Myeloma, Myeloma

Correction to: *Leukemia* 10.1038/s41375-023-01928-7, published online 17 May 2023

In this article, Ryoji Ito at affiliation Central Institute for Experimental Animals, Kanagawa, Japan was missing from the author list.

The original article has been corrected.

