# Supplementary material for: UTX inactivation in germinal center B cells promotes the development of multiple myeloma with extramedullary disease
Source: Leukemia. 2023 May 17;37(9):1895–907. doi: 10.1038/s41375-023-01928-7 (PMC10457198; doi:10.1038/s41375-023-01928-7)
Supplement: Supplementary file 1 — Supplementary Information [file 41375_2023_1928_MOESM1_ESM.pdf]

Utx inactivation in germinal center B cells promotes the development of multiple myeloma with extramedullary disease

Ola Rizq[1,2,3,4]*, Naoya Mimura[3,5*#], Motohiko Oshima[1,2], Shuji Momose[6], Naoya Takayama[7], Naoki Itokawa[1], Shuhei Koide[1], Asuka Shibamiya,[3] Yurie Miyamoto-Nagai[3], Mohamed Rizk[1,2], Yaeko Nakajima-Takagi[1,2], Kazumasa Aoyama[1,2], Changshan Wang[2,8], Atsunori Saraya[2], Masanori Seimiya[9], Mariko Watanabe[10], Satoshi Yamasaki[11], Tatsuhiro Shibata[11], Kiyoshi Yamaguchi[12], Yoichi Furukawa[12], Tetsuhiro Chiba[13], Emiko Sakaida[3,5], Chiaki Nakaseko[14], Jun-ichi Tamaru[6], Yu-Tzu Tai[4], Kenneth C. Anderson[4], Hiroaki Honda[15], and Atsushi Iwama[1,2,16#]

Supplementary Information:

Supplementary Materials and Methods

Supplementary References

Supplementary Figure 1-5

Supplementary Materials and Methods

Establishment of multiple myeloma cell line BU749

Total ascitic fluid cells (77% PCs by flow cytometry) were harvested from a moribund *Utx*$^{\Delta/\Delta}$ *Braf* ^{V600E} female mouse and rinsed in PBS containing FBS (Sigma-Aldrich). The cells were resuspended in RPMI-1640 supplemented with 10% fetal bovine serum (FBS) (Sigma-Aldrich), 1% penicillin/streptomycin/glutamine (Gibco), 1 mM Sodium pyruvate (11360-070, Gibco), 0.1 mM NEAA (MEM NEAA, 11140-050, Gibco), 5x10^{-5} M 2-mercaptoethanol (Sigma-Aldrich), 10 ng/ml mouse recombinant IL-6 (575706**,** Biolegend), and co-cultured with/without Tst4 cells.

Conditional expression of *UTX*

BU749 plasma cells were transduced with the all-in-one Tet-on inducible lentiviral vector (Ai-LV) for the expression of human *UTX*.[1] Transduced cells were purified by cell sorting using GFP as a marker. *UTX* expression was induced by the addition of doxycycline (2 µg/ml). Lentiviral vectors expressing wild-type *UTX* or an enzymatically dead *UTX* (H1146A and E1148) were prepared as previously described.[2,3] The UTX-delcIDR cDNA[4] (kindly provided by Dr. Hao Jiang, University of Virginia School of Medicine, Charlottesville, VA) was subcloned into CSII-IRES-Venus lentivirus vector. Recombinant lentiviruses were produced using established protocols[5].

Cell culture and drug treatment

Human MM cells MM1.S, NCI-H929 (H929), RPMI8226, and U266 cell lines were obtained from American Type Culture Collection. Human ARD cells[6-8] were a kind gift of Dr. Jonathan D. Licht (The University of Florida Health Cancer Center, Gainesville, FL). They were

maintained in RPMI-1640 supplemented with 10% FBS and 1% penicillin/streptomycin/glutamine. Bortezomib (Selleck Chemicals), JQ-1 (Sigma-Aldrich), and lenalidomide (Selleck Chemicals) were diluted in dimethyl sulfoxide (DMSO; Sigma-Aldrich).

Flow Cytometry and Fluorescence-Activated Cell Sorting

For surface flow cytometry and cell sorting of mouse BM, spleen, enlarged lymph nodes, tumors, or body fluids, single-cell suspensions were obtained according to standard protocols. Cell populations were analyzed using a BD FACS Canto II and/or sorted using BD FACSAria IIu or III (BD Biosciences, San Jose, CA, USA). Data were analyzed using FlowJo software (Tree Star). The antibodies used for flow cytometric analysis are shown below.

Antibody	Source	Clone	Catalogue number
PE anti-mouse CD138	BioLegend	281-2	142503
PE anti-mouse CD138	Miltenyi Biotec	REA104	130-102-580
Purified anti-mouse CD16/32	BioLegend	93	101301
Biotin anti-mouse/human CD11b	TONBO Biosciences	M1/70	30-0112-U025
PE anti-mouse/human CD11b	BioLegend	M1/70	101207
APC anti-mouse/human CD11b	BioLegend	M1/70	101211
VioletFluor 450 anti-mouse CD45.2	TONBO Biosciences	104	75-0454-U025
FITC anti-mouse CD45.1	BioLegend	A20	110705
PE anti-mouse Ly-6G/Ly-6C (Gr1)	BioLegend	RB6-8C5	108407
APC anti-human/mouse CD45R/B220	TONBO Biosciences	RA3-6B2	20-0452-U025
FITC anti-mouse/human CD45R/B220	BioLegend	RA3-6B2	103205
PE/Cy7 anti-mouse/human CD45R/B220	BioLegend	RA3-6B2	103221
APC/Cy7 anti-mouse CD4	BioLegend	RM4-5	100525
APC anti-mouse CD4	BioLegend	GK1.5	100411
APC/Cy7 anti-mouse CD8a	BioLegend	53-6.7	100713
APC anti-mouse CD8a	BioLegend	53-6.7	100711
PE anti-mouse CD23	BioLegend	B3B4	101607
APC anti-mouse Ig light chain λ	BioLegend	RML-42	407306
PE-Cy7 Rat Anti-Mouse Ig, κ Light Chain	BD Biosciences	187.1	560667
Biotin anti-mouse IgA Antibody	BioLegend	RMA-1	407003
APC/Cy7 anti-mouse IgD	BioLegend	11-26c.2a	405715
FITC Rat Anti-Mouse IgG1	BD Biosciences	A85-1	553443
FITC anti-mouse IgM	BioLegend	RMM-1	406505

PE anti-mouse/human GL7 Antigen	BioLegend	GL7	144607
APC anti-Mouse CD95 (APO-1/Fas)	eBioscience	15A7	17-0951
APC anti-mouse CD21/CD35 (CR2/CR1)	BioLegend	7E9	123411
APC/Cy7 Streptavidin	BioLegend		405208

Immunoblot analysis

Whole-cell lysates were prepared by lysis in RIPA (50 mM Tris, pH 8.0, 150 mM NaCl, 1mM EDTA, pH 8.0,1% TritonX-100, 0.1% sodium deoxycholate and 0.1% SDS) or PML (20 mM sodium phosphate, pH 7.0, 300 mM NaCl, 5 mM EDTA and 0.1% NP40) buffers supplemented with protease inhibitor cocktail (Roche), or SDS-sample buffer (25 mM Tris, pH 6.8, 1% SDS, 5% glycerol, 0.05% bromophenol blue and 1% β-mercaptoethanol). Lysates were then sonicated (Bioruptor, COSMO BIO CO.) prior to SDS-PAGE. Immunoblotting was performed according to standard procedures. Membranes were probed with the indicated antibodies to: H3 (Abcam, ab1791), H3K27me3 (Millipore, 07449), UTX (D3Q1I, Cell Signaling, 33510) GAPDH (clone 14C10, Cell Signaling, 2118), α-tubulin (Calbiochem, CP06), anti-flag (clone M2, Sigma-Aldrich, F1804), or H3K27ac (Abcam, ab4729). HRP-conjugated secondary antibodies were purchased from Amersham ECL. Immobilon Western Chemiluminescent substrates (EMD Millipore) were used for immunoblot detection. Sequential re-probing of the membranes was performed after stripping of primary and secondary antibodies using 62.5mM Tris, pH 6.8, 2% SDS and 0.7% 2-mercaptoethanol. Protein expression was quantified using Image Lab software (BioRad).

Assays of cytotoxicity

Cell lines were dissociated, counted, and plated in flat-bottom tissue culture 96-well plates (TPP). Human cell lines were plated at 8,000-20,000 cells per well and cultured with the indicated doses. For MTS assay, CellTiter 96 AQueous One Solution (Promega) was added to the cells in the last four hours of the incubation period and absorbance was read on a plate

reader (TriStar2, LB942, BERTHOLD, Bad Wildbad, Germany) to determine relative cell number in each well. Data were averaged for triplicates or quadruplicates and normalized to the untreated wells. Results are expressed as the percentage of untreated control.

Quantitative RT-PCR

Total RNA was isolated using RNeasy Plus Micro (Qiagen). cDNA was made using the ThermoScript RT-PCR system (Invitrogen) with an oligo-dT primer. Real-time quantitative PCR was performed in triplicate using TB Green Premix Ex Taq II (Tli RNaseH Plus) (Takara Bio) on a StepOnePlus Real-Time PCR System (Applied Biosystems). The real-time PCR signals were examined in triplicates and normalized to those of *Beta-actin* (*Actb*) gene. The primer sequences used are listed as follows (all 5′-3′):

Myc, Forward: CTGCTGTCCTCCGAGTCCT, Reverse: GCCTCTTCTCCACAGACACC;

Actb, Forward: CTAAGGCCAACCGTGAAAAG, Reverse: ACCAGAGGCATACAGGGACA.

PCR analysis of *Igh* and *Igκ* rearrangement

Sorted cells were resuspended in 1x buffer (50 mM Tris, pH 8.0, 20 mM EDTA, 0.5% SDS) supplemented with proteinase K (Roche), and DNA was isolated by phenol extraction and ethanol precipitation. *Igh* and *Igκ* rearrangements were amplified by PCR using the following primers:

DSF, AGGGATCCTTGTGAAGGGATCTACTACTGTG

J_H4, AAAGACCTGCAGAGGCCATTCTTACC

DQ52, GCGGAGCACCACAGTGCAACTGGGAC

Vκ, GGCTGCAGSTTCAGTGGCAGTGGRTCWGGRAC

Jκ5, ATGCGACGTCAACTGATAATGAGCCCTCTCC

PCR products were separated on agarose gels and stained by ethidium bromide.

RNA sequencing

Total RNA was purified from 0.3 – 1.0 × 10^4 BM plasma cells or in vitro cultured murine cells using the RNeasy plus Micro Kit (QIAGEN). RNA concentration and integrity were verified using Agilent 2100 Bioanalyzer (Santa Clara, CA, USA). Amplification, construction of the libraries and sequencing were performed as previously described.[9] Hisat2 (version 2.1.0) and Bowtie2 (version 2.3.4.3) were used for alignment to the reference mouse genome (mm10 from the University of California, Santa Cruz Genome Browser; http://genome.ucsc.edu/) using the annotation data from iGenomes (Illumina) with default parameters. Normalization and significant expression differences were detected using DESeq2 (version 2.2.1)[10] with raw counts generated from StringTie (version 1.3.4). Transcripts Per Million (TPM) values were also calculated using StringTie (version 1.3.4).

Chromatin immunoprecipitation (ChIP) sequencing and CUT&TAG of histone modifications

ChIP-seq and CUT&TAG of histone modifications were performed using a previously described protocol[11].

Assay for transposase-accessible chromatin with high-throughput sequencing (ATAC seq) and data processing

A transposase reaction was performed using nuclei prepared from 7,500 freshly sorted cells. Libraries were generated using a NEBNext Ultra DNA Library Prep Kit (New England BioLabs, Beverly, MA, USA). Library DNA was size-selected (240–360 bps) using BluePippin (Sage Science, Beverly, MA, USA). Sequencing was performed using HiSeq 2500 (Illumina)

with a single-read sequencing length of 60 bp. Sequences were aligned to mouse genome sequences (mm10) using Bowtie2 (default setting). Mapped reads were subsampled using samtools to make the numbers of reads in all samples the same. Total read counts in each sample were subsampled to match the smallest one and standardized in library-size as count per million (CPM). In addition, CPMs were also corrected in the step of calculation of differentially accessible regions (DARs) by DESeq2 using the DESeq2 internal method called the median-of-ratios method, which is similar to the trimmed mean of M values method (TMM).[10] Macs2 (version 2.2.6) was used to call peaks using nomodel, a narrow peak option. Using a q-value cutoff of 0.001, accessible peaks were detected in each sample The catalogue of all peaks called in any samples was produced by merging all called peaks that overlapped by, at least, one base pair using the Bedtools merge function. The Bedtools map function was used to count the reads at each region in the catalog using bed files of each sample. Read count matrix of each sample was used for detection of DARs by using DESeq2. For the heatmap, normalized read counts obtained using DESeq2 were z-score-scaled and plotted. For motif analysis, findMotifsGenome.pl of Homer was used with the -size200-mask option. For the annotation of peaks, annotatePeaks.pl of Homer was used with the default settings. For visualization, RPM values of the sequenced reads were calculated for every 200-base pair bin with a shifting size of 100 base pairs by using bed tools, and then converted to a bigWig file using the wigToBigWig tool.

Gene set enrichment analysis (GSEA)

Gene set enrichment analysis was conducted with the software GSEA (http://www.broadinstitute.org/gsea).[12] A pseudocount of 1 was added to the TPM (transcripts per million) measure prior to GSEA.

Whole exome sequencing (WES) and mutation calling

CD138$^+$ transformed plasma cells were purified by cell sorting from BM, spleen, or ascitic fluid. PB B cells and tail were used as controls. Whole-exome capture libraries were prepared from extracted DNA, using a SureSelect Mouse All Exon kit (Agilent Technologies, Santa Clara, CA, USA) according to the manufacturer's protocol. In brief, 10 - 60 ng of DNA was fragmented using a Covaris S220 system (Covaris Inc. Woburn, MA, USA) to produce fragments with an average size of 150–200 base pairs, followed by end repair, A-tailing, and ligation. Pre-capture polymerase chain reaction (PCR) amplification of the adapter-ligated library was performed for 9 or 11 cycles depending on the starting amount using SureSelect XT HS Index Primers (Agilent Technologies). The adapter-ligated library was purified using Agencourt AMPure XP beads (Beckman Coulter, Brea, CA, USA). Quantity and the size distribution of the library were confirmed using TapeStation 2200 (Agilent Technologies). Next, 500 - 1000 ng of the amplified libraries were hybridized using SureSelect Mouse All Exon kit (Agilent Technologies), and purified using Dynabeads MyOne Streptavidin T1 (Thermo Fisher Scientific). Post-capture amplification was performed for 9 cycles. Purification of amplified libraries was performed with Agencourt AMPure XP beads (Beckman Coulter, Brea, CA, USA). Quantity and size distribution of the amplified libraries were determined using TapeStation 2200 (Agilent Technologies) and KAPA Library Quantification Kit (KAPA Biosystems). Prepared libraries were sequenced in a paired-end mode, using HiSeq 2500 platform (Illumina Inc., San Diego, CA, USA). All sequenced reads were aligned to the mouse reference genome (GRCm38) using the BWA-MEM program[13] as paired-end reads. Probable PCR duplications, in which paired-end reads aligned to the same genomic positions, were removed, and pile-up files were generated using SAMtools[14] and a program developed in house. The single nucleotide variations (SNV) and short insertions/deletions (indel) detected in target and control (PB B cell or tail) samples were compared to filter somatic mutations, in the same

manner as our previously reported work.[15] Details of filtering conditions are provided in Supplementary Table 5B.

Accession numbers

RNA- and ChIP-sequencing data and CUT&TAG data obtained in this study were deposited in DNA Data Bank of Japan (DDBJ) (accession numbers DRA13765 and DRA015180).

Peripheral blood cell count analysis

Blood cell count measurement was performed on a Celltac Alpha VET MEK-6550 analyzer (Nihon Kohden, Japan).

Histological analysis of mouse tissue

All mouse tissues were fixed in 10% buffered formalin and embedded in paraffin. The tissue blocks were cut into 3-μm sections and stained with hematoxylin-eosin staining for histopathological analysis. For immunohistochemistry, 3-μm sections were stained with the antibodies listed below on an automated immunostainer Leica Bond III (Leica Microsystems, Buffalo Grove, IL, USA).

Antibodies	Source	Clone	Catalogue number
Anti-B220	abcam	RA3-6B2	ab64100
Anti-Bcl6	abcam	7D1	ab243150
Anti-CD138	BD Biosciences	281-2	553712
Anti-Pax5	abcam	EPR3730(2)	ab109443
Anti-CD3	BioRad	CD3-12	MCA1477

Serum protein electrophoresis (SPEP)

Mice were bled by tail grazing shortly before sacrifice. Blood samples were spun at 2,000×g for 10 minutes to collect serum. Serum was loaded into quick gel SP (Helena Laboratories, Beaumont, TX, USA) and separated by electrophoresis (Epalyzer 2, Helena Laboratories). The gels were stained with ponceau S (M3) (Helena Laboratories) and then destained with 2% acetic acid.

Statistical analysis

Statistical significance of difference was measured by unpaired 2-tailed Student's t test or Welch's test when the variance was judged as significantly different. P values less than 0.05 were considered significant, using Graph Pad Prism, version 4. Survival was assessed using Kaplan–Meier curves and log-rank analysis (Graph Pad Prism, version 9.4).

Supplementary References

1. Yamaguchi T, Hamanaka S, Kamiya A, et al. Development of an All-in-One Inducible Lentiviral Vector for Gene Specific Analysis of Reprogramming. *PLoS One*. 2012;7(7):e41007. doi:10.1371/journal.pone.0041007
2. Sengoku T, Yokoyama S. Structural basis for histone H3 Lys 27 demethylation by UTX/KDM6A. *Genes & Development*. 2011;25(21):2266-2277.
3. Hong S, Cho Y-W, Yu L-R, Yu H, Veenstra TD, Ge K. Identification of JmjC domain-containing UTX and JMJD3 as histone H3 lysine 27 demethylases. *Proc Natl Acad Sci U S A*. 2007;104(47):18439-18444.
4. Shi B, Li W, Song Y, et al. UTX condensation underlies its tumour-suppressive activity. *Nature*. 2021;597(7878):726-731.
5. Iwama A, Oguro H, Negishi M, et al. Enhanced Self-Renewal of Hematopoietic Stem Cells Mediated by the Polycomb Gene Product Bmi-1. *Immunity*. 2004;21(6):843-851.
6. Hardin J, MacLeod S, Grigorieva I, et al. Interleukin-6 Prevents Dexamethasone-Induced Myeloma Cell Death. *Blood*. 1994/11/01/ 1994;84(9):3063-3070.
7. Ridley RC, Xiao H, Hata H, Woodliff J, Epstein J, Sanderson RD. Expression of Syndecan Regulates Human Myeloma Plasmα Cell Adhesion to Type I Collagen. *Blood*. 1993/02/01/ 1993;81(3):767-774.
8. Ezponda T, Dupéré-Richer D, Will CM, et al. UTX/KDM6A Loss Enhances the Malignant Phenotype of Multiple Myeloma and Sensitizes Cells to EZH2 inhibition. *Cell Rep*. 21(3):628-640. doi:10.1016/j.celrep.2017.09.078

9. Mochizuki-Kashio M, Aoyama K, Sashida G, et al. Ezh2 loss in hematopoietic stem cells predisposes mice to develop heterogeneous malignancies in an Ezh1-dependent manner. *Blood*. 2015;126(10):1172-1183.
10. Love MI, Huber W, Anders S. Moderated estimation of fold change and dispersion for RNA-seq data with DESeq2. *Genome Biology*. 2014;15(12):550.
11. Itokawa N, Oshima M, Koide S, et al. Epigenetic traits inscribed in chromatin accessibility in aged hematopoietic stem cells. *Nat Commun*. 2022/05/16 2022;13(1):2691.
12. Subramanian A, Tamayo P, Mootha VK, et al. Gene set enrichment analysis: A knowledge-based approach for interpreting genome-wide expression profiles. *Proceedings of the National Academy of Sciences*. 2005;102(43):15545-15550.
13. Li H, Durbin R. Fast and accurate short read alignment with Burrows–Wheeler transform. *Bioinformatics*. 2009;25(14):1754-1760.
14. Li H, Handsaker B, Wysoker A, et al. The Sequence Alignment/Map format and SAMtools. *Bioinformatics*. 2009;25(16):2078-2079.
15. Totoki Y, Tatsuno K, Covington KR, et al. Trans-ancestry mutational landscape of hepatocellular carcinoma genomes. *Nat Genet*. 2014;46(12):1267-1273.

A

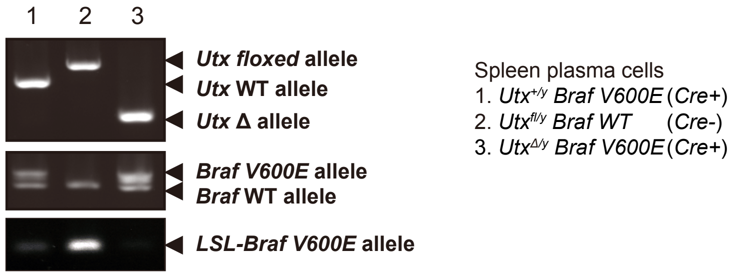

B

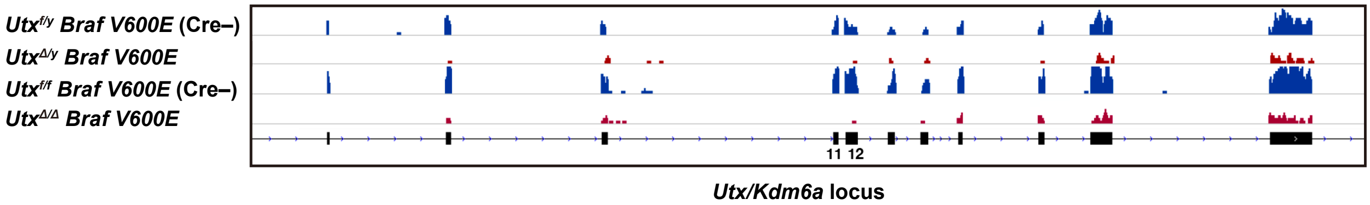

Supplementary Figure 1. Targeting of the *Utx* gene in the mouse hematopoietic system
(A) Efficient deletion of *Utx* exons 11 and 12 detected by genomic PCR in spleen plasma cells from the indicated mice. Floxed, floxed *Utx* allele; Δ, floxed *Utx* allele after the removal of exons 11 and 12 by Cre recombinase.
(B) A snapshot of genome browser tracks of RNA-seq signals at *Utx* locus in BM plasma cells from the indicated mice after immunization.

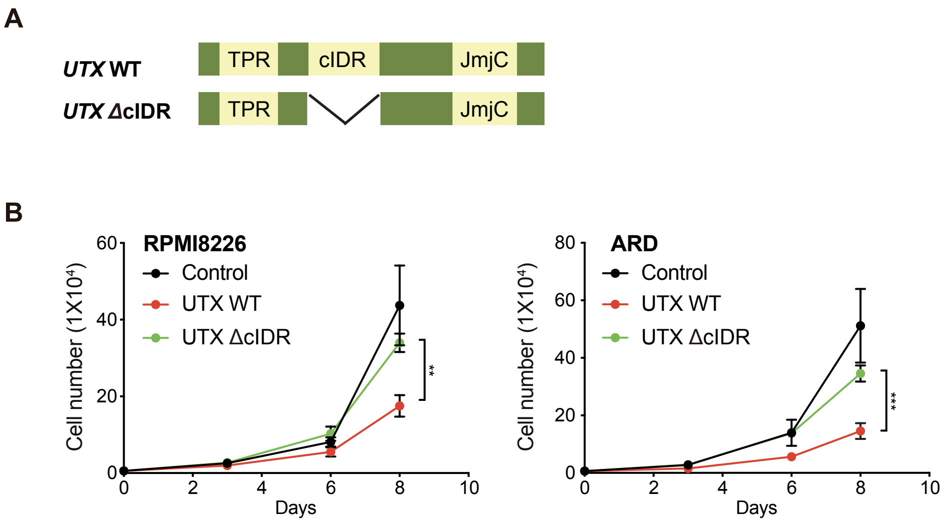

Supplementary Figure 2. A UTX mutant lacking cIDR region shows significant loss of function in human UTX-null MM cells
(A) Schematic representation of WT and ΔcIDR UTX.
(B) Effects of WT and ΔcIDR UTX add-back on UTX-null human MM RPMI8226 and ARD cell growth.
Cell growth data are shown as the mean ± SD of triplicate cultures. Statistical significance of cell growth was determined by the student *t*-test. **, $P < 0.01$; ***, $P < 0.001$.

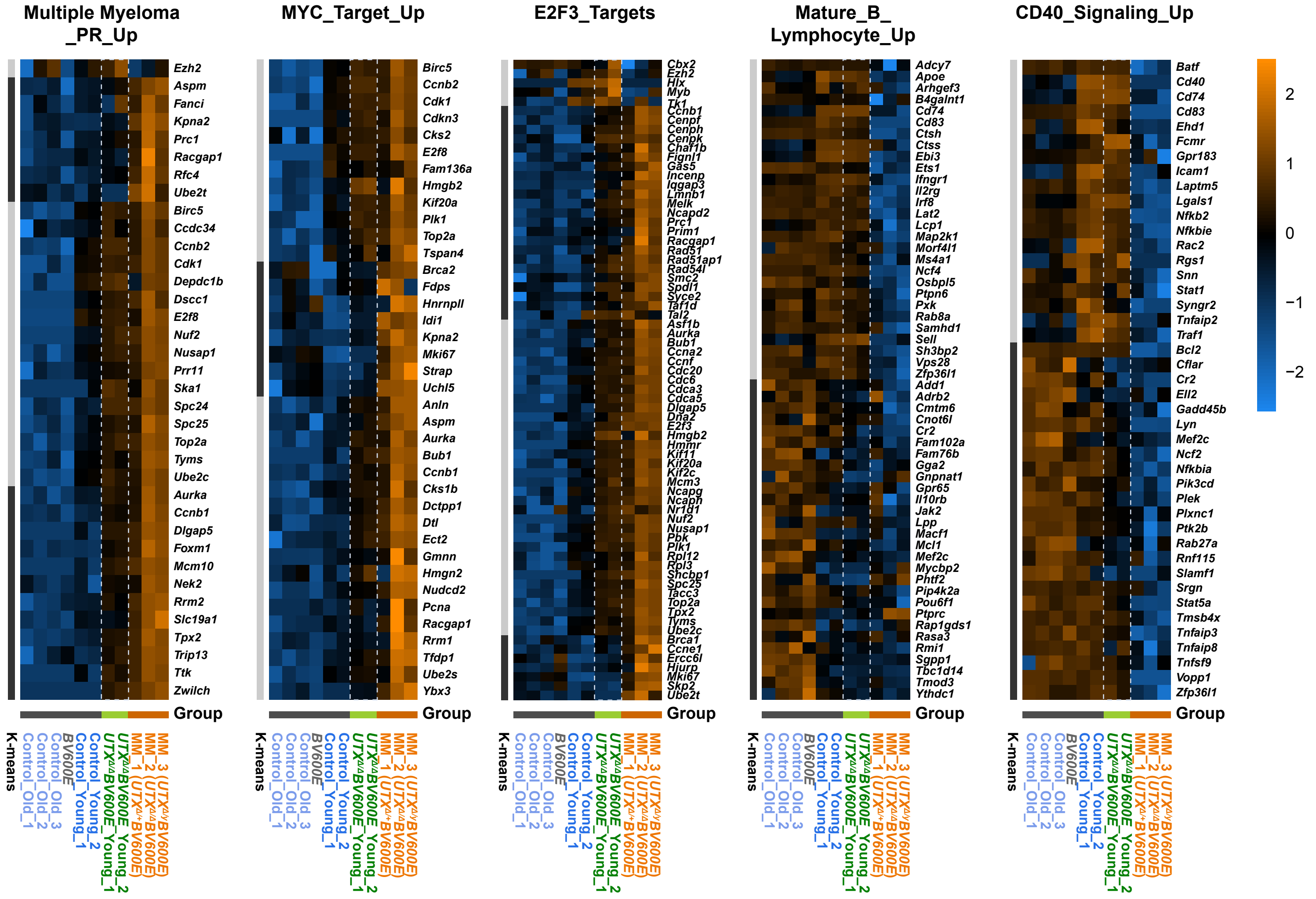

Supplementary Figure 3 Transcriptomic profiles of representative gene sets associated with multiple myeloma
K-means clustering of RNA-seq data.
The representative gene sets shown are ZHAN_MULTIPLE_MYELOMA_PR_UP; YU_MYC_TARGETS_UP; KONG_E2F3_TARGETS; MORI_MATURE_B_LYMPHOCYTE_UP; and BASSO_CD40_SIGNALING_UP.

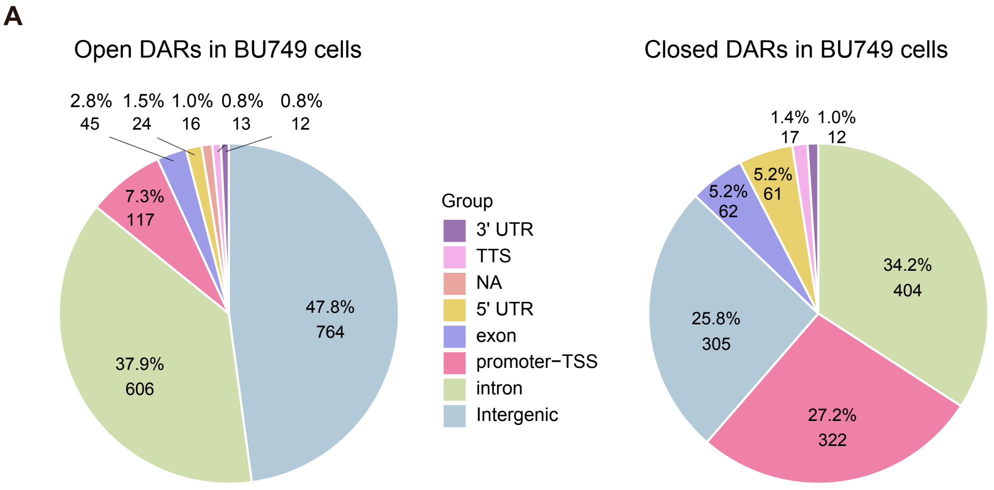

B

	All regions		Open DARs	
	Total peaks	With motif	Total peaks	With motif
PU.1-IRF	57639	9998 (17%)	2661	880 (33%)
PU.1:IRF8	57639	3944 (7%)	2661	409 (15%)
PU.1	57639	7229 (13%)	2661	623 (23%)
SpiB	57639	4284 (7%)	2661	419 (16%)

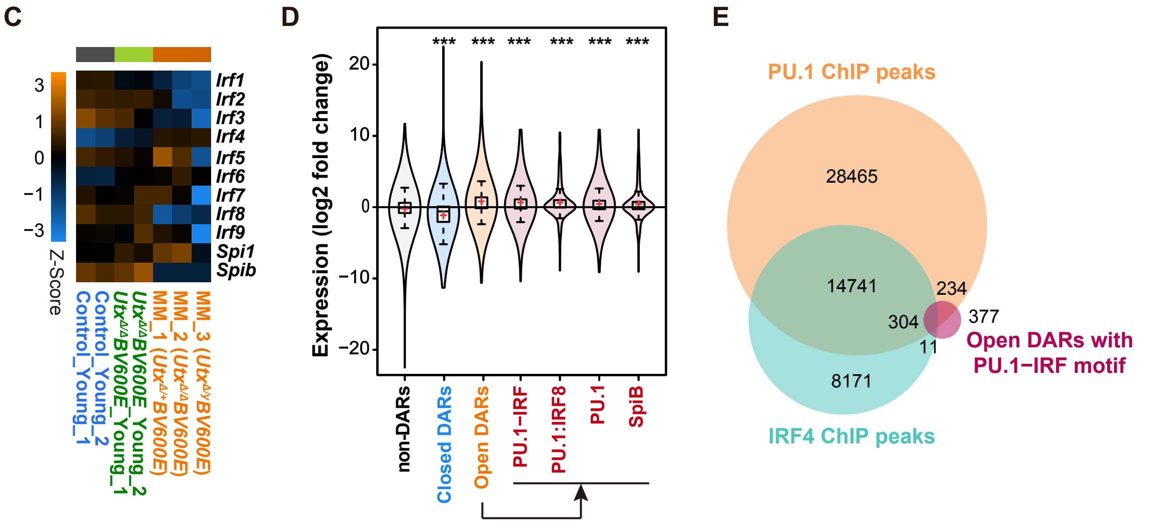

Supplementary Figure 4. Chromatin accessibility in BU749 cells

(A) Pie charts showing the percentage of each annotation of all accessible peaks.

(B) Motif analysis of open DARs in BU749 cells. Motifs with $p<10^{-10}$, log2FC > 1 are depicted. The enrichment of each motif in open DARs and the background peaks are indicated. All peaks, which include peaks from all fractions, were used as background.

(C) Heatmap showing the z-scores of expression values (DESeq2 normalized counts) of *Irf* genes, *Spi1*, and *Spib* from RNA-seq data.

(D) Violin plots showing the expression of genes linked to open and closed DARs in BU749 cells. Expression in BU749 cells relative to control plasma cells is depicted. Expression of genes linked to the open DARs with indicated transcription factor binding motifs and genes linked to non-DARs is also indicated.

(E) Venn diagram showing the overlap between DARs with PU.1-IRF motif, and the regions bound by IRF4 or PU.1 in ChIP-seq analysis (retrieved from published data: Minnich et al. Nature Immunology. 2016; 17(3):331-343).

A

CC50

Cell Line		BTZ (nM)	Lenalidomide (μM)	JQ1 (μM)
MM1.S	(UTX WT)	1.9	45.8	4.1
H929	(UTX WT)	1.8	74.3	2.6
RPMI8226	(UTX null)	4.5	377.6	21.5
U266	(UTX mut)	3.1	309.0	64.3

B

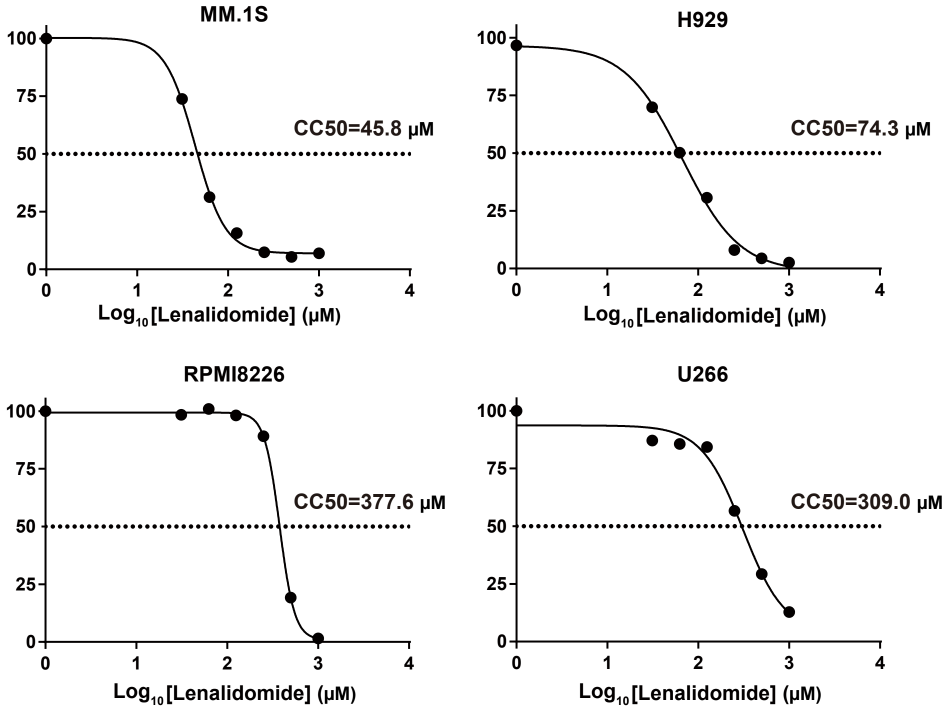

C

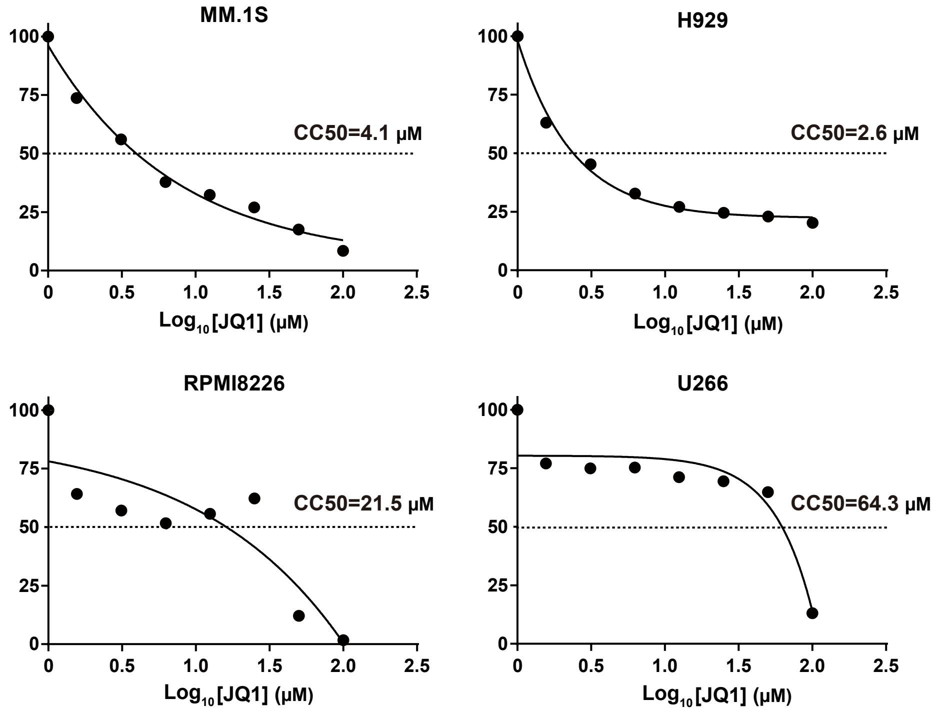

Supplementary Figure 5. CC50 of bortezomib, lenalidomide, and JQ1 in MM cell lines
(A) Summary of CC50 of bortezomib (BTZ), lenalidomide, and JQ1 in MM cell lines.
(B, C) CC50 plots of MM cells. Cells were treated with the indicated concentrations of lenalidomide or JQ1 for 72h or 48 h, respectively, in triplicate. CC50 was defined as the concentration required to reduce cell viability by 50%. Cell viability was determined by MTS assays.
